# Glycine Supplementation Enhances the Growth of Sow-Reared Piglets with Intrauterine Growth Restriction

**DOI:** 10.3390/ani15131855

**Published:** 2025-06-23

**Authors:** Shengdi Hu, David W. Long, Fuller W. Bazer, Robert C. Burghardt, Gregory A. Johnson, Guoyao Wu

**Affiliations:** 1 Department of Animal Science, Texas A&M University, College Station, TX 77843, USA; 2 Department of Veterinary Integrative Biosciences, Texas A&M University, College Station, TX 77843, USA; rburghardt@cvm.tamu.edu (R.C.B.);

**Keywords:** amino acids, glycine, growth, IUGR piglets, milk, nutrition, protein synthesis

## Abstract

Glycine has the greatest rate of deposition in whole-body proteins among all amino acids in neonates, but its provision from sow’s milk meets only 20% of the requirement of suckling piglets. The results of our recent studies indicate that piglets with intrauterine growth restriction (IUGR) have a reduced ability to synthesize glycine. The present study determined the role of glycine in the growth of sow-reared IUGR piglets. In Experiment 1, 56 newborn piglets (postnatal day 0) with a low birth weight (<1.10 kg) were selected from 14 litters, providing 4 IUGR piglets/litter that were allotted randomly into one of four treatment groups (14 piglets/group). Piglets received oral administration of either 0, 0.1, 0.2 or 0.4 g glycine/kg body weight (BW) twice daily (i.e., 0, 0.2, 0.4 or 0.8 g glycine/kg BW/day) between 0 and 14 days of age. L-Alanine was used as the isonitrogenous control. The BWs of all piglets were recorded each week during the experiment. Two weeks after the initiation of glycine supplementation, blood and tissue samples were collected for biochemical analyses. In Experiment 2, rates of muscle protein synthesis in tissues were determined on day 14 using the ^3^H-phenylalanine flooding dose technique. Compared with piglets in the control group, oral administration of 0.2, 0.4 and 0.8 g glycine/kg BW/day did not affect their milk intake (*p* > 0.05) but increased (*p* < 0.05) concentrations of glycine in plasma by 1.52-, 1.94-, and 2.34-fold, respectively, and body weight by 20%, 37%, and 34%, respectively. The dose of 0.4 g glycine/kg BW/day was the most cost-effective. Consistent with its growth-promoting effect, glycine supplementation stimulated (*p* < 0.05) the phosphorylation of mechanistic target of rapamycin (MTOR), eukaryotic initiation factor 4E binding protein 1 (4E-BP1), and ribosomal protein S6 kinase beta-1 (p70^S6K^) as well as protein synthesis in skeletal muscle, compared with the control group. Collectively, oral administration of glycine activated the MTOR signaling pathway in skeletal muscle and enhanced the growth performance of IUGR piglets. These results indicate that endogenous synthesis of glycine is inadequate to meet the needs of IUGR piglets during the suckling period and that oral supplementation with glycine to these compromized neonates can improve their growth performance.

## Simple Summary

Intrauterine growth restriction (IUGR) is a significant problem in swine nutrition, affecting up to 25% of piglets with Duroc, Hampshire, Landrace, and Yorkshire genetic backgrounds (having a live-born litter size of 10–13) and ~30% of piglets from prolific dams with an average live-born litter size of sows of ~18. Due to the lack of proper nutritional intervention, IUGR piglets are culled at birth on most swine farms, representing a major loss to the global pork industry and a concern over animal welfare. The results of this study indicate that oral administration of glycine (0.2, 0.4 and 0.8 g/kg body weight per day) to sow-reared IUGR piglets enhanced the availabilities of glycine, glutathione (a major antioxidant), and creatine (essential for energy metabolism in muscle and brain) in tissues, as well as growth performance, while reducing the concentrations of ammonia, urea, and oxidants in plasma. Glycine supplementation did not affect the circulating levels of cortisol, insulin, growth hormone, or insulin-like growth factor I, but stimulated protein synthesis in tissues (including skeletal muscle). Thus, glycine is a conditionally essential amino acid for sow-reared IUGR piglets and can improve their growth and the profit of swine production.

## 1. Introduction

Glycine has the greatest rate of accretion among all amino acids in both mammalian fetuses and neonates [1]. Thus, dietary requirements of growing animals for glycine are particularly high [2]. This is consistent with the multiple roles of glycine in cell signaling, metabolism [e.g., the synthesis of creatine and glutathione (GSH)], and functions including activation of the mechanistic target of rapamycin (MTOR) cell signaling pathway in cells [3,4,5,6]. However, glycine has traditionally been classified as a “nutritionally non-essential amino acid” for mammals including humans and pigs [7,8,9,10], because it can be synthesized from serine [11], threonine [12], choline [13], and 4-hydroxyproline [14,15] in a tissue-specific manner. However, emerging evidence indicates that glycine from sow’s milk provides only 20% of the requirement of suckling piglets and that de novo synthesis may be inadequate for their maximal growth [2,16,17]. Therefore, glycine should be considered as a “conditionally essential amino acid” for the optimal growth, development, and health of animals, particularly neonates.

Intrauterine growth restriction (IUGR), defined as fetal or birth weight less than two standard deviations below the mean body weight (BW) for breed and gestational age [18,19], is a significant problem in mammals, including swine. The mean BW of live-born piglets at birth is 1.4 kg for the offspring of Yorkshire × Landrace sows bred with Duroc × Hampshire boars; a birth weight of <1.1 kg is considered as IUGR [20]. Up to 25% of live-born piglets with Duroc, Hampshire, Landrace, and Yorkshire genetic backgrounds (with a litter size of 10–13) are affected by IUGR [20,21,22,23]. More newborn piglets (e.g., ~30% of all live-born piglets) from prolific dams with an average live-born litter size of sows of ~18 may exhibit IUGR [24,25,26]. IUGR negatively affects the postnatal survival, growth, development, body composition, feed efficiency, health, and productivity of offspring [27,28,29,30,31,32,33,34,35,36], as compared to littermates with a normal birth weight (NBW) [37]. Thus, IUGR piglets are often culled at birth, resulting in not only substantial losses to the pork industry but also concerns over animal welfare [38,39].

Wang et al. [17] found that adding 1–2% glycine (on a dry matter basis) to a liquid milk diet promoted lean tissue growth in NBW piglets. Most recently, we reported that dietary supplementation with 1% glycine to IUGR pigs beginning from weaning (21 days of age) until they reached market weight (~120 kg BW) enhanced their growth performance [40]. Whether sow-reared IUGR piglets respond positively to glycine supplementation is unknown because they may respond differently to the same nutrient (e.g., leucine or protein) than NBW piglets [41,42,43]. For example, oral administration of L-leucine at a dose of 200% of its intake from milk reduced BW gain and body protein accretion in neonatal piglets with IUGR but had positive effects in those with NBWs [41,42]. In addition, increasing protein intake by 50% promotes muscle growth in NBW piglets but causes high rates of morbidity and mortality in IUGR piglets [43]. Clearly, specific nutritional means should be designed for IUGR neonates.

The objective of this study was to test the hypothesis that oral administration of glycine to sow-reared IUGR piglets improves their growth performance. The pig was chosen because it is a species of agricultural significance and a useful model in human nutrition research [44,45,46].

## 2. Materials and Methods

### 2.1. Experiment 1

Piglets were the offspring of Yorkshire × Landrace female swine (parities 1–4) bred with Duroc boars. The average number of live-born piglets was 12 per litter. Throughout the lactation period, sows had free access to drinking water, as well as a corn- and soybean meal-based diet (Table 1) that adequately provided all nutrients recommended by the National Research Council [47]. The birth weights of all newborn piglets (day 0 of age) were recorded immediately after farrowing. A total of 56 IUGR piglets (birth weight < 1.1 kg; 0.83 ± 0.02 kg, mean ± SEM, n = 56) were selected from 14 litters, providing 4 IUGR piglets/litter that were assigned randomly into one of four treatment groups (14 piglets/group) to receive oral administration of either 0, 0.2, 0.4, or 0.8 g glycine/kg BW/day. All IUGR piglets exhibited the characteristics of a steep dolphin-like forehead and bulging eyes (Supplementary Figure S1). At the start of the experiment, there was an equal number of female and male piglets in each treatment group. All animals were maintained at the Texas A&M University’s Swine Center.

#### 2.1.1. Oral Administration of Glycine

Beginning on the day of birth (day 0), immediately after nursing, piglets received oral administration of either 0, 0.1, 0.2, or 0.4 g glycine/kg BW twice daily (8:00 AM and 5:00 PM) for 14 days, which were defined as Groups 1, 2, 3, and 4, respectively. These doses were chosen on the basis of results from a previous study involving early-weaned piglets fed milk protein-based diets with or without glycine [17]. L-Alanine was used as the isonitrogenous control [17,19]. Namely, pigs in the 0, 0.1, 0.2, and 0.4 g glycine/kg BW twice daily groups received oral administration of 0.475, 0.356, 0.237, and 0 g L-alanine /kg BW twice daily, respectively. Glycine or alanine was dissolved in 10 mL distilled and deionized water before gavaging. Piglets were weighed at 0, 7 and 14 days of age. During the experimental period, 4 piglets died in each group (2 males and 2 females in Group 1, 2 males and 2 females in Group 2, 3 males and 1 female in Group 3, and 2 males and 2 females in Group 4). Thus, at the end of the experiment, there were 10 piglets in each treatment group. The ages at mortality were 3.3 ± 1.7, 4.5 ± 2.0, 6.5 ± 1.9, and 7.8 ± 2.1 days (means ± SEM, n = 4 pigs; *p* = 0.393) in the 0, 0.2, 0.4, and 0.8 g glycine/kg BW/day groups, respectively.

#### 2.1.2. Determination of Milk Consumption by Piglets

On day 13 of the experiment, the milk consumption of piglets was determined between 09:00 and 20:00 using the weigh–suckle–weigh method as previously described [48]. Briefly, piglets were initially removed from their mothers at 09:00 and returned to their mothers at 11:00 for 1 h nursing (defined as one meal). Piglets were removed from their mothers at 12:00 and returned to their mothers at 13:00 for 1 h nursing; this same procedure was performed until 20:00 (the last measurement). Each IUGR piglet in a litter was weighed before and after each of the five meals to calculate its milk intake.

#### 2.1.3. Collection of Blood Samples

On day 14 of the experiment, 6 piglets (3 males and 3 females) in each group were selected randomly and fasted for 1.5 h. Jugular vein blood samples (5 mL) were withdrawn from each animal into an EDTA-containing tube, a heparinized tube, and a plain tube. An aliquot (0.2 mL) of whole blood in the heparinized tube was mixed with 0.2 mL of 0.225% iodoacetate (an alkylating agent to preserve GSH) containing 0.05% sodium heparin, 0.5% serine, and 100 mM sodium borate. The blood samples were centrifuged immediately at 10,000× *g* for 1 min, and the supernatant fluid (plasma from the EDTA-containing tube, plasma from the iodoacetate tube, and serum from the plain tube) was stored at −80 °C until analyzed.

#### 2.1.4. Collection of Skeletal Muscle and Other Tissues from Piglets

On day 14 of the experiment, immediately after blood collection, piglets were euthanized by intra-cardiac administration of saturated KCl following anesthesia with an intramuscular injection of Telazol (10 mg/kg BW). A sample (~5 g) of longissimus lumborum muscle (formerly known as longissimus dorsi muscle) on the left side of the pig was obtained rapidly, frozen in liquid nitrogen, and stored at −80 °C. Liver, small intestine, stomach, pancreas, heart, and kidneys were also collected and weighed.

#### 2.1.5. Analysis of Amino Acids in Plasma and Tissues

Concentrations of amino acids in plasma and tissues were determined using high-performance liquid chromatography (HPLC) methods, as we described previously [48]. Briefly, 0.1 mL of plasma was mixed with 0.1 mL of 1.5 M HClO_4_, followed by the addition of 2.25 mL HPLC-grade H_2_O and 0.05 mL of 2 M K_2_CO_3_ to the tube (the tube was immersed in ice). Approximately 100 mg of tissue was homogenized with 1 mL of 1.5 M HClO_4_, followed by the addition of 5 mL HPLC-grade H_2_O and 0.5 mL of 2 M K_2_CO_3_. The neutralized solution was centrifuged at 600× *g* and 4 °C for 10 min, and the supernatant fluid was used directly for the analysis of all amino acids except proline and cysteine using an HPLC method involving precolumn derivatization with 30 mM *o*-phthaldialdehyde (OPA). Proline was measured after its oxidation to 4-amino-1-butanol, followed by precolumn derivatization with OPA. For determinations of cysteine, a 100 µL sample was mixed with 50 µL of 50 mM iodoacetic acid for 5 min at 25 °C for the formation of *S*-carboxymethylcysteine, followed by precolumn derivatization with OPA. Amino acids were quantified on the basis of authentic standards (Sigma Chemicals, St. Louis, MO, USA) using the Millennium^TM^ workstation (Waters Inc., Milford, MA, USA).

#### 2.1.6. Analysis of Glucose, Urea, Ammonia, and Hormones in Plasma or Serum

Concentrations of glucose, urea, and ammonia in plasma were analyzed using hexokinase, urease, and glutamate dehydrogenase, respectively [19]. Concentrations of free fatty acids, triacylglycerols, and total cholesterol in plasma were determined using the assay kits Cat. #994-75409 (Wako Chemicals, Richmond, VA, USA), Cat. #2780-250 (Thermo DMA, Louisville, CO, USA), and Cat. #2350-250 (Thermo DMA), respectively. Thiobarbituric acid reactive substances (TBARS, an indicator of malondialdehyde (a product of lipid peroxidation [49])) in plasma were quantified using a kit (Cat # 10009055) from Cayman Chemical (Ann Arbor, MI, USA). Concentrations of total cortisol in plasma, as well as insulin, growth hormone, and insulin-like growth factor-1 (IGF-1) in serum, were determined, as we described previously [19]. The employed assay kits for porcine hormones were Cat. #TKCO-1 (Diagnostic Products, Los Angeles, CA, USA), Cat. #PI-12K (Linco, St. Charles, MO, USA), Cat. #PGH-46HK (Linco), and Cat. #DSL-10-2800 (Diagnostic Systems Laboratories, Inc., Webster, TX, USA), respectively.

#### 2.1.7. Analysis of Creatine, Phosphocreatine, Creatinine, and Guanidinoacetate

Concentrations of creatine, phosphocreatine, creatinine, and guanidinoacetate in plasma and tissues were analyzed by HPLC [50]. Briefly, plasma (100 µL) was mixed with an equal volume of 1.5 M HClO_4_, followed by the addition of 50 µL of 2 M K_2_CO_2_. Approximately ~100 mg of frozen tissue was homogenized in 1 mL of 1.5 M HClO_4_, followed by the addition of 1 mL of HPLC-grade H_2_O and 0.5 mL of 2 M K_2_CO_2_. The neutralized solution was centrifuged at 600× *g* and 4 °C for 10 min to obtain the supernatant fluid for analysis. For the determination of creatine (not subject to boiling), 20 µL of a sample (or creatine standard) plus 80 µL of HPLC-grade water was mixed with 30 mM benzoin (5 µL), 100 mM β-mercaptoethanol plus 200 mM sodium sulfite (5 µL), and 2 M KOH (10 µL) in an autosampler. Following separation on a Supelco C18 column by a gradient of 0.1 M sodium acetate/HPLC-grade methanol, the creatine–benzoin derivative was detected at excitation and emission wavelengths of 325 and 425 nm, respectively. Creatinine and guanidinoacetate in samples were analyzed as described previously, after being boiled for 15 min under alkaline conditions (converting creatinine into creatine). Phosphocreatine was determined after a sample (50 µL) was incubated at 37 °C for 30 min with 10 µL of creatine kinase (1 mg/mL) and 50 µL of 10 mM ADP plus 100 mM MgCl_2_ to convert phosphocreatine into creatine.

#### 2.1.8. Analysis of Reduced Glutathione (GSH) and Oxidized Glutathione (GSSG)

Concentrations of GSH and GSSG in plasma and tissues were determined using an HPLC method involving precolumn derivatization with OPA [50]. Briefly, plasma (0.2 mL) was mixed with 0.1 mL of Reagent B (a mixture of 1.24 g boric acid, 12.9 mL of 70% HClO_4_, and 97.1 mL HPLC-grade H_2_O) and then with 50 μL of 2 M K_2_CO_3_. Frozen tissue (~100 mg) was homogenized with 3 mL of a mixture of 1.5 M HClO_4_ and 12 mM iodoacetate (1:1, *vol*/*vol*) and then neutralized with 0.75 mL of 2 M K_2_CO_3_. Neutralized solutions were analyzed for GSH and GSSG. For the determination of GSH, a 50 µL sample was mixed with 50 µL of 25 mM iodoacetate for 10 min at 25 °C, followed by reaction with OPA to form a fluorescent product for detection at excitation and emission wavelengths of 340 and 450 nm, respectively. For the analysis of GSSG, a 50 µL sample was reacted with 100 µL of 28 mM 2-mercaptoethanol (a reducing agent) at 25 °C for 5 min, resulting in the formation of GSH, which was quantified as described previously.

#### 2.1.9. Western Blot Analysis

Western blot analyses of proteins in skeletal muscle were performed as we described previously [51]. Briefly, frozen tissue was ground to powder under liquid nitrogen, followed by homogenization with a lysis buffer [20 mM Tris-HCl (pH 7.4), 50 mM NaCl, 50 mM NaF, 50 mM of EDTA, 1% Triton X-100, 1× protease inhibitor cocktail, and 1× phosphatase inhibitor cocktail] (Calbiochem, La Jolla, CA, USA). Proteins in homogenates were quantified using the bicinchoninic acid assay method. Thereafter, the samples were diluted with a 2× Laemmli buffer (125 mM Tris-HCl pH 6.8, 4% *w*/*v* SDS, 10% 2-mercaptoethanol, 12% glycerol, and 0.004% *w*/*v* bromphenol blue) and boiled for 5 min. Samples (50 µg protein) were separated on 4–12% SDS-polyacrylamide gels (Bio-Rad, Hercules, CA, USA), followed by the transfer of proteins onto a nitrocellulose membrane under 12 V overnight. The membranes were blocked in 5% fat-free dry milk in TTBS (20 mM Tris/150 mM NaCl, pH 7.5, and 0.1% Tween-20) for 3 h and then incubated overnight at 4 °C with gentle rocking with one of the primary antibodies for target proteins. They were MTOR (Cell Signaling, Danvers, MA, USA; 1:1000), phosphorylated MTOR (Cell Signaling, 1:1000), eukaryotic initiation factor 4E binding protein 1 (4E-BP1; Cell Signaling, 1:1000), phosphorylated 4E-BP1 (Cell Signaling, 1:1000), ribosomal protein S6 kinase beta-1 (p70^S6K^; Cell Signaling, 1:1000), phosphorylated p70^S6K^ (Cell Signaling, 1:1000), and glyceraldehyde-3-phosphate dehydrogenase [GAPDH (a house-keeping protein); Cell Signaling, 1:1000]. After being washed three times with TTBS, the membranes were incubated at 25 °C for 2–3 h with a secondary antibody at a dilution of 1:50,000 (peroxidase-labeled donkey anti-goat or anti-rabbit IgG, Jackson Immuno Research, West Grove, PA, USA). After the membranes were washed with TTBS, images of the target proteins were developed using the Super Signal West Dura Extended Duration Substrate (Pierce, Rockford, IL, USA), with the signals being detected on Fujifilm LAS-3000 (Tokyo, Japan).

### 2.2. Experiment 2

This experiment was conducted as described above, except that two IUGR piglets were selected from each of 12 litters and then assigned randomly into one of two treatment groups (12 piglets/group) to receive oral administration of either 0 or 0.4 g glycine/kg BW/day. The birth weight (< 1.1 kg) of IUGR piglets was 0.85 ± 0.03 kg (mean ± SEM, n = 24). During the experimental period, 4 piglets (2 males and 2 females) died in each group. Thus, at the end of the experiment, there were 8 piglets in each treatment group for the measurement of protein synthesis in tissues. The ages at mortality were 3.8 ± 1.5 and 7.3 ± 1.8 days (means ± SEM, n = 4 piglets; *p* = 0.186) in the 0 and 0.4 g glycine/kg BW/day groups, respectively.

On day 14 of the experiment, each piglet received an intraperitoneal administration of 10 mL [^3^H]phenylalanine solution per kg BW (150 µmol of L-phenylalanine, 3.5 µCi of L-[ring-2,4-^3^H]phenylalanine, and 100 µmol NaCl per mL; pH 7.0), which was equivalent to 1.5 mmol phenylalanine and 35 µCi [^3^H]phenylalanine per kg BW. Thirty minutes after the intraperitoneal administration of the [^3^H]phenylalanine solution, the piglets received an intramuscular injection of Telazol (10 mg/kg BW) and then an intra-cardiac injection of 40 mL of saturated KCl for euthanasia. Thereafter, samples (~5 g) of longissimus lumborum muscle (on the left side of the piglet), gastrocnemius muscle (on the right side of the piglet), liver, jejunum, and kidney were obtained immediately, frozen in liquid nitrogen, and stored at –80 °C. Thereafter, the longissimus lumborum muscle (between the 3rd and 7th ribs on the right side of the piglet), gastrocnemius muscle (on the right side of the piglet), liver, jejunum, and kidney were obtained and weighed.

The specific radioactivities of [^3^H]phenylalanine in tissue protein and the intramuscular free pool were determined as described previously [52]. Briefly, a tissue sample (~0.55 g) was homogenized in 2 mL of 2% trichloroacetic acid (TCA). The homogenate was transferred to a glass tube (resistant to 200 °C), and the homogenizer was rinsed with 2 mL of 2% TCA. The combined homogenates (4 mL) were centrifuged (3000× *g* for 15 min), and the supernatant (TCA-soluble fraction) was analyzed for phenylalanine and ^3^H-phenylalanine (i.e., the specific radioactivity of free phenylalanine). The protein pellet (TCA-insoluble fraction) was washed with 5 mL of 2% TCA three times, and the protein pellet was hydrolyzed in 6 mL of 6 M HCl at 110 °C for 24 h under N_2_. The protein hydrolysates were analyzed for phenylalanine and ^3^H-phenylalanine (i.e., the specific radioactivity of protein-bound phenylalanine). The fractional rate of protein synthesis (%/day) in tissue was calculated as (S_b_ ÷ S_a_) × T ÷ t, where S_b_ is the specific radioactivity of protein-bound ^3^H-phenylalanine, S_a_ is the specific radioactivity of free ^3^H-phenylalanine, T is 1440 min/d, and t is the time of ^3^H-phenylalanine labeling. The labeling time was 30 min for liver and longissimus lumborum muscle, 31 min for the jejunum, and 32 min for the gastrocnemius muscle and kidneys. The absolute rate of protein synthesis (g/day) was calculated as the amount of protein × the fractional rate of protein synthesis [52].

### 2.3. Statistical Analysis

Results are expressed as means ± SEM. All data were first tested for normality using the Shapiro–Wilk W Test in the JMP 15 Pro software (Cary, NC, USA), and their normal distribution was confirmed by a probability of >0.05. Within each treatment group, there were no differences (*p* > 0.05) in growth or any measured metabolites between male and female piglets based on the unpaired *t*-test. Thus, sex was not included as a variable in the statistical analysis. Growth data (BW and weight gain) were analyzed using two-way analysis of variance, with glycine and day as two factors. Metabolic data (obtained only at one time point) in Experiment 1 were analyzed using one-way analysis of variance [53]. Differences among treatment means were determined using the Student–Newman–Keuls multiple comparison test. Data on tissue protein synthesis in Experiment 2 were analyzed using the unpaired *t*-test. A probability (*p*) value of ≤0.05 was taken to indicate statistical significance.

## 3. Results

### 3.1. Experiment 1

#### 3.1.1. Milk Consumption of Piglets

The consumption of milk by IUGR piglets measured at 13 days of age was 236 ± 15, 228 ± 12, 244 ± 18, and 240 ± 16 mL/kg BW/day, respectively, in the 0, 0.2, 0.4, and 0.8 g glycine/kg BW/day groups, with a mean value of 237 mL/kg BW/day. Increasing glycine supplementation from 0 to 0.8 g/kg BW/day did not affect (*p* > 0.05) milk intake by IUGR piglets. Based on the content of glycine in sow’s whole milk (e.g., 1.12 g/L) [54], the average intake of glycine by IUGR piglets was 265 mg/kg BW/day.

#### 3.1.2. Body Weights and Weight Gains of Piglets

The effects of oral administration of glycine to IUGR piglets on their growth are summarized in Table 2. At 7 days of age, the BWs of surviving piglets in the 0.2, 0.4, and 0.8 g glycine/kg BW/day groups did not differ (*p* > 0.05) from each other but were 15–27% greater (*p* < 0.05) than those in the control group. At 14 days of age, the BWs of surviving piglets increased (*p* < 0.05) by 20–37% in a dose-dependent manner as the supplemental amount of glycine increased from 0 to 0.4 g glycine/kg BW/day, but the BWs of piglets did not differ (*p* > 0.05) between the 0.4 and 0.8 g glycine/kg BW/day groups. Weight gains between 7 and 0, between 14 and 7, and between 14 and 0 days of age increased (*p* < 0.05) by 30–58%, 31–63%, and 31–60%, respectively, in a dose-dependent manner as the supplemental amount of glycine increased from 0 to 0.4 g glycine/kg BW/day, but weight gains during each age period did not differ (*p* > 0.05) between the 0.4 and 0.8 g glycine/kg BW/day groups.

#### 3.1.3. Tissue Weights of Piglets

Glycine supplementation (0 to 0.4 g/kg BW/day) to IUGR piglets increased the absolute weights of the longissimus lumborum muscle, gastrocnemius muscle, small intestine, liver, kidneys, pancreas, stomach, and heart in a dose-dependent manner (Table 3). However, the weight of each of these tissues did not differ (*p* > 0.05) between the 0.4 and 0.8 g glycine/kg BW/day groups. The relative weight of each tissue in IUGR piglets (calculated as the percentage of BW) was not affected (*p* > 0.05) by glycine supplementation.

#### 3.1.4. Concentrations of Amino Acids in Plasma

Glycine supplementation increased the concentrations of glycine and serine in the plasma of IUGR piglets (Table 4). Specifically, as compared with the control group, supplementation with 0.2, 0.4, and 0.8 g glycine/kg BW/day increased concentrations of glycine in plasma in a dose-dependent manner, by 1.52-, 1.94-, and 2.34-fold (*p* < 0.05), respectively. Compared with the control group, concentrations of serine increased (*p* < 0.05) by 18% and 25%, respectively, in the plasma of piglets receiving oral administration of 0.4 and 0.8 g glycine/kg BW/day. Concentrations of alanine in the plasma of piglets without glycine supplementation (with the administration of an isonitrogenous amount of alanine) were 44–47% greater (*p* < 0.05) than those in the 0.2, 0.4, and 0.8 g glycine/kg BW/day groups. Concentrations of other amino acids in plasma (including arginine, aspartate, branched-chain amino acids, glutamate, histidine, hydroxyproline, lysine, methionine, ornithine, and proline) did not differ (*p* > 0.05) among the four groups of piglets (Table 4).

#### 3.1.5. Concentrations of Amino Acids in Tissues

Compared to the control group, the oral administration of 0.2, 0.4, and 0.8 g glycine/kg BW/day to IUGR piglets dose-dependently increased (*p* < 0.05) concentrations of glycine in the longissimus lumborum muscle, gastrocnemius muscle, liver, small intestine, and kidneys by 25–88%, 32–98%, 20–64%, 22–73%, and 15–65%, respectively (Table 5). Increasing the doses of glycine supplementation from 0 to 0.2 and to 0.4 g/kg BW/day increased (*p* < 0.05) the concentrations of serine by 13–22% and 12–23% in the longissimus lumborum muscle and gastrocnemius muscle (Table 5), respectively. Concentrations of serine were 27%, 22%, and 19% greater (*p* < 0.05) in the liver, small intestine, and kidneys, respectively, of piglets receiving the oral administration of 0.4 g glycine/kg BW/day, as compared to the control group, but did not differ (*p* > 0.05) either between the control and the 0.2 g glycine/kg BW/day groups or among the 0.2, 0.4, and 0.8 g glycine/kg BW/day groups (Table 5). In all the tissues examined, concentrations of other amino acids (including alanine, aspartate, glutamate, and glutamine) did not differ (*p* > 0.05) among the four groups of piglets (Supplementary Tables S1–S5).

#### 3.1.6. Concentrations of Glucose, Nitrogenous Metabolites, Lipids, TBARS, and Hormones in Plasma or Serum

Concentrations of glucose, free fatty acids, and total cholesterol in the plasma of IUGR piglets were not affected (*p* > 0.05) by oral administration of 0.2–0.8 g glycine/kg BW/day, compared to the control group (Table 6). Increasing glycine supplementation from 0 to 0.2 and 0.4 g/kg BW/day decreased (*p* < 0.001) concentrations of ammonia in plasma by 12% and 25%, respectively, and those of urea by 10% and 22%, respectively (Table 6). Concentrations of ammonia or urea in plasma did not differ (*p* > 0.05) between the 0.4 and 0.8 g glycine/kg BW/day groups. Compared with the control group, the oral administration of 0.2, 0.4, and 0.8 g glycine to IUGR piglets dose-dependently reduced concentrations of TBARS in plasma by 11%, 23%, and 33%, respectively (Table 6). Glycine supplementation did not influence (*p* > 0.05) concentrations of cortisol in plasma or concentrations of insulin, growth hormone, or insulin-like growth factor-I (IGF-I) in serum (Table 6).

#### 3.1.7. Concentrations of Creatine, Phosphocreatine, Creatinine, and Guanidinoacetate in Plasma and Tissues

Data on concentrations of creatine and related substances in the plasma and tissues of IUGR piglets are summarized in Table 7. Compared to the control group, oral administration of 0.2 and 0.4 g glycine/kg BW/day to IUGR piglets dose-dependently increased (*p* < 0.05) concentrations of creatine, phosphocreatine, and creatine plus phosphocreatine in plasma, longissimus lumborum muscle, gastrocnemius muscle, jejunum, and kidneys by 17–30%, 15–28%, 14–31%, 13–31%, and 16–33%, respectively. Increasing the doses of glycine supplementation from 0 to 0.2 and to 0.4 g/kg BW/day increased (*p* < 0.05) concentrations of creatine by 16% and 31% in liver, respectively, without affecting those of phosphocreatine. Compared to the control group, oral administration of 0.2 and 0.4 g glycine/kg BW/day to IUGR piglets dose-dependently increased (*p* < 0.05) concentrations of guanidinoacetate in plasma and kidneys by 14–29%, and 15–31%, respectively, without affecting those in the longissimus lumborum muscle, gastrocnemius muscle, liver, and jejunum. In plasma and all the tissues examined, concentrations of creatine and related substances did not differ (*p* > 0.05) between the 0.4 and 0.8 g/kg BW/day groups.

#### 3.1.8. Concentrations of GSH and GSSG in Plasma and Tissues

Effects of glycine supplementation on concentrations of GSH and GSSG in the plasma and tissues of sow-reared IUGR piglets are summarized in Table 8. Concentrations of GSSG in plasma, longissimus lumborum muscle, gastrocnemius muscle, liver, jejunum, and kidney did not differ (*p* > 0.05) among piglets receiving the oral administration of 0, 0.2, and 0.4 g glycine/kg BW/day, but were decreased (*p* < 0.05) by 17%, 25%, 23%, 20%, 18%, and 23%, respectively, in the 0.8 g glycine/kg BW/day group as compared to the control group. Compared to the control group, oral administration of 0.2, 0.4, and 0.8 g glycine/kg BW/day to IUGR piglets dose-dependently increased (*p* < 0.05) concentrations of GSH in plasma, liver, and jejunum by 13–46%, 14–47%, and 23–71%, respectively, while decreasing (*p* < 0.05) GSSG/GSH ratios by 17–43%, 21–45%, and 24–53%, respectively. Concentrations of GSH in the longissimus lumborum muscle and kidney did not differ (*p* > 0.05) among piglets receiving oral administration of 0 and 0.2 g glycine/kg BW/day, but were increased (*p* < 0.05) by 32–50%, 27–44%, and 20–31%, respectively, in the 0.4–0.8 g glycine/kg BW/day groups as compared to the control group. Increasing the doses of glycine supplementation from 0 to 0.2, 0.4, and 0.8 g/kg BW/day dose-dependently decreased (*p* < 0.05) GSSG/GSH ratios by 20–49%, 17–46%, and 17–41%, respectively, in the longissimus lumborum muscle and kidney.

### 3.2. Experiment 2

#### 3.2.1. Rates of Protein Synthesis in Tissues

In Experiment 2, the initial BWs of surviving IUGR piglets at day 0 of age were 0.92 ± 0.02 and 0.93 ± 0.03 kg (means ± SEM, n = 8; *p* = 0.770) in the 0 and 0.4 g glycine/kg BW/day groups, respectively; the final BWs of the surviving piglets at day 14 of age were 2.52 ± 0.07 and 3.38 ± 0.10 kg (means ± SEM, n = 8; *p* < 0.001) in the 0 and 0.4 g glycine/kg BW/day groups, respectively. Increasing the oral administration of glycine from 0 to 0.4 g/kg BW/day increased (*p* < 0.05) the fractional rates of protein synthesis in the longissimus lumborum muscle, gastrocnemius muscle, liver, small intestine, and kidneys by 16%, 15%, 17%, 18%, and 16%, respectively (Table 9). Because the amounts of protein per tissue in all of the studied tissues were greater (*p* < 0.01) in glycine-supplemented than in control piglets, the absolute rates of protein synthesis were also greater (*p* < 0.01) in the former than in the latter (Table 9). Specifically, increasing the oral administration of glycine from 0 to 0.4 g/kg BW/day increased (*p* < 0.05) the absolute rates of protein synthesis in the longissimus lumborum muscle, gastrocnemius muscle, liver, small intestine, and kidneys by 50%, 53%, 52%, 56%, and 54%, respectively.

#### 3.2.2. Proteins in the MTOR Cell Signaling Pathway in Skeletal Muscle

**Figure 1:**
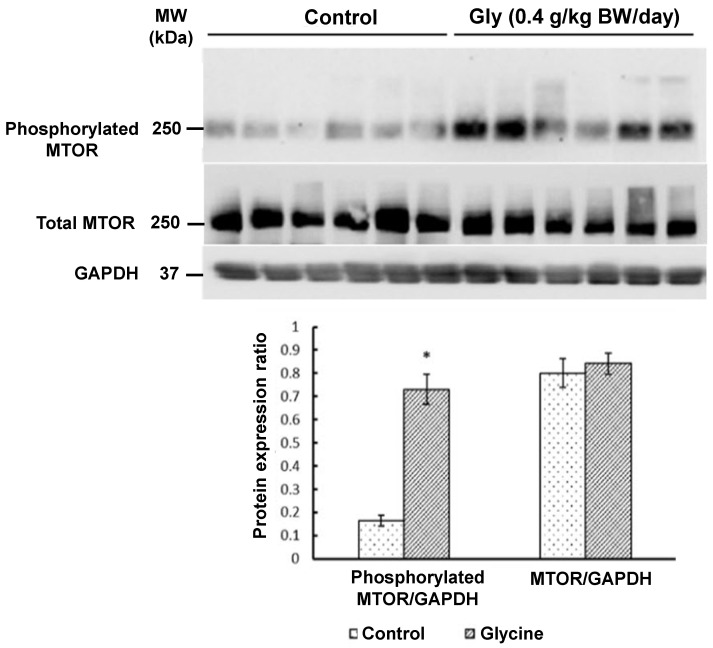
Abundances of total mechanistic target of rapamycin (MTOR) and phosphorylated MTOR proteins in the skeletal muscle of IUGR piglets with or without glycine supplementation. Newborn piglets (postnatal day 0) with low birth weights were allotted randomly into one of four treatment groups. Piglets received oral administration of either 0 or 0.4 g glycine/kg body weight/day) between 0 and 14 days of age. Alanine was used as the isonitrogenous control. At 14 days of age, skeletal muscle (longissimus lumborum muscle) was obtained for Western blot analyses of proteins. Glyceraldehyde-3-phosphate dehydrogenase (GAPDH) protein was used as the internal control. Values are means ± SEM, n = 6 per group. Data were analyzed by one-way ANOVA and the Student–Newman–Keuls multiple comparison test. * *p* < 0.05: different from the control group (*p* < 0.05). The molecular weights (MWs) of the measured proteins are indicated.

**Figure 2:**
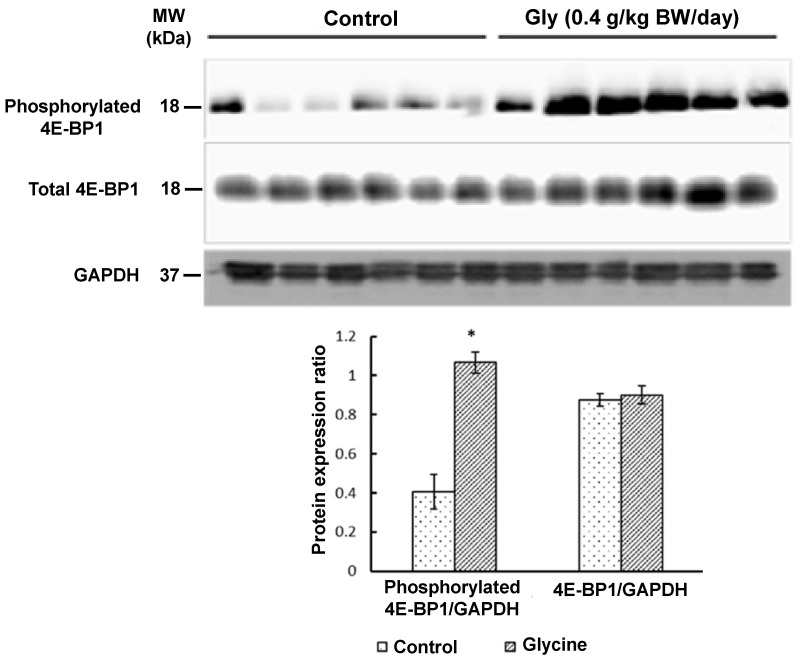
Abundances of total eukaryotic initiation factor 4E binding protein 1 (4E-BP1) and phosphorylated 4E-BP1 proteins in skeletal muscle of IUGR piglets with or without glycine supplementation. Newborn piglets (postnatal day 0) with low birth weights were allotted randomly into one of four treatment groups. Piglets received oral administration of either 0 or 0.4 g glycine/kg body weight/day) between 0 and 14 days of age. Alanine was used as the isonitrogenous control. At 14 days of age, skeletal muscle (longissimus lumborum muscle) was obtained for Western blot analyses of proteins. Glyceraldehyde-3-phosphate dehydrogenase (GAPDH) protein was used as the internal control. Values are means ± SEM, n = 6 per group. Data were analyzed by one-way ANOVA and the Student–Newman–Keuls multiple comparison test. * *p* < 0.05: different from the control group (*p* < 0.05). The molecular weights (MWs) of the measured proteins are indicated.

**Figure 3:**
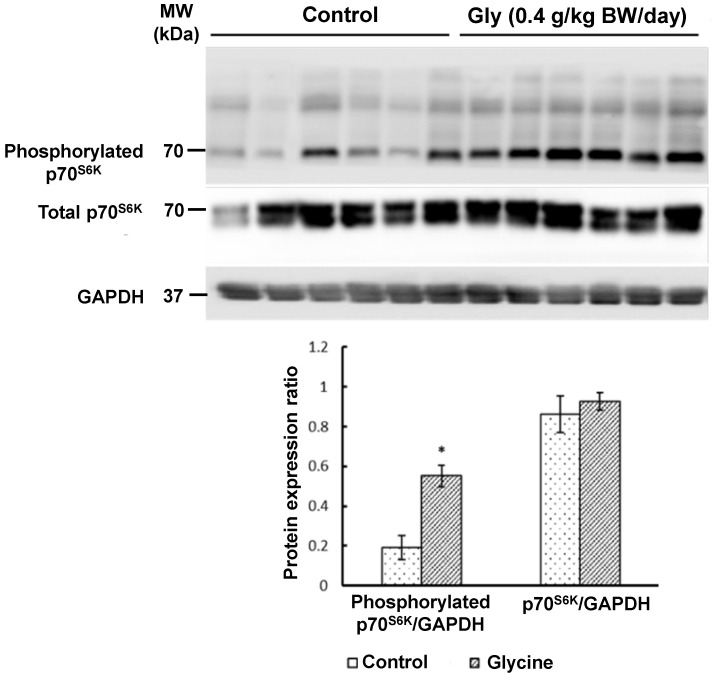
Abundances of total ribosomal protein S6 kinase beta-1 (p70^S6K^) and phosphorylated p70^S6K^ proteins in skeletal muscle of IUGR piglets with or without glycine supplementation. Newborn piglets (postnatal day 0) with low birth weights were allotted randomly into one of four treatment groups. Piglets received oral administration of either 0 or 0.4 g glycine/kg body weight/day) between 0 and 14 days of age. Alanine was used as the isonitrogenous control. At 14 days of age, skeletal muscle (longissimus lumborum muscle) was obtained for Western blot analyses of proteins. Glyceraldehyde-3-phosphate dehydrogenase (GAPDH) protein was used as the internal control. Values are means ± SEM, n = 6 per group. Data were analyzed by one-way ANOVA and the Student–Newman–Keuls multiple comparison test. * *p* < 0.05: different from the control group (*p* < 0.05). The molecular weights (MWs) of the measured proteins are indicated.

Western blot analyses revealed that glycine supplementation increased (*p* < 0.05) the abundance of phosphorylated MTOR (Figure 1), phosphorylated p70^S6K^ (Figure 2), and phosphorylated 4E-BP1 (Figure 3) in the longissimus lumborum muscle of IUGR piglets. Specifically, the expression of phosphorylated MTOR was 330% greater (*p* < 0.01) in piglets receiving 0.4 g glycine/kg BW/day than in control piglets (Figure 1). The 4E-BP1 and p70^S6K^ proteins in the muscle had 170% and 180% greater (*p* < 0.05) levels of phosphorylation, respectively, in response to glycine supplementation (Figure 2 and Figure 3). In contrast, the abundances of total MTOR, total p70^S6K^, or total 4E-BP1 proteins in the muscle did not differ (*p* > 0.05) between the two groups of piglets (Figure 1, Figure 2 and Figure 3).

## 4. Discussion

Glycine has versatile roles in the nutrition, metabolism, and general health of animals [5]. It has been classified as a “non-essential amino acid” for mammals, but this term is now recognized as a misnomer [55]. Several lines of evidence have documented the beneficial effects of glycine on the maintenance and growth of mammals with NBWs. First, glycine regulates the expression and distribution of mucosal barrier proteins (claudin-7 and ZO-3) in intestinal epithelial cells [56]. Second, glycine stimulates protein synthesis and inhibits proteolysis in skeletal muscle [57], which is of enormous importance in both swine production and human health. The underlying mechanisms involve the activation of MTOR cell signaling [57,58,59,60,61] and enhanced protein synthesis, as well as improved antioxidative responses and the reduced expression of pro-inflammatory cytokines [60,62,63,64]. Third, dietary supplementation with glycine enhances the growth performance and feed efficiency of milk-fed NBW piglets [17] and of postweaning piglets with either NBWs or IUGR [40]. To our knowledge, this is the first report regarding the beneficial effects of glycine supplementation on the growth performance of sow-reared IUGR piglets.

Glycine is deficient in unsupplemented IUGR piglets, as its concentration in plasma is only half of that in age-matched NBW piglets on postnatal days 1 and 7 of age [16]. Insufficient endogenous synthesis of glycine may contribute, in part, to growth restriction in IUGR neonates [2]. A novel and important finding from this work is that oral administration of glycine (0.2–0.8 g/kg BW/day) to IUGR piglets enhanced their growth, with the dose of 0.4 g glycine/kg BW/day being the most cost-effective (Table 3). As indicated previously, oral administration of leucine (1.4 g/kg and 2.1 g/kg BW twice daily) to 7-day-old preweaning IUGR piglets for 2 weeks reduced their rate of growth [42], and supplementation with high doses of milk protein caused deaths of IUGR piglets [43] likely due to ammonia toxicity. In contrast, these compromised neonates responded to the oral administration of glycine with an improvement of BW and lean tissue gains (Table 4). It is possible that most of the supplemental glycine is used for whole-body protein synthesis and, therefore, only a small amount of glycine is oxidized to generate ammonia in the piglets. This is consistent with a relatively low rate of whole-body glycine oxidation to CO_2_ in piglets [2]. Hence, there was no evidence of toxicity in IUGR pigs receiving dietary supplementation with up to 0.8 g/kg BW/day. In this regard, glycine may offer an advantage over leucine or protein as a nutritional supplement to IUGR piglets, although both amino acids can activate MTOR cell signaling and protein synthesis in skeletal muscle [60,65,66,67,68,69]. Our results also provide clear evidence in support of the notion that endogenous synthesis of glycine is insufficient for maximal growth and feed efficiency in IUGR piglets.

Glycine is essential for the synthesis of creatine and glutathione [70,71,72,73,74,75,76,77,78]. Our findings that the concentrations of creatine and phosphocreatine as well as GSH were greater in the plasma, skeletal muscle, small intestine, liver, and kidneys of glycine-supplemented IUGR pigs than those in unsupplemented littermates (Table 8) indicate insufficient endogenous synthesis of glycine. Interestingly, compared to the 0.4 g glycine/kg BW/day group, supplementing 0.8 g glycine/kg BW/day to sow-reared IUGR piglets increased concentrations of GSH, but not creatine and phosphocreatine, in all the tissues examined (Table 7), suggesting that intracellular concentrations of glutamate and cysteine are sufficient for GSH synthesis but those of arginine [19], methionine [74], or both, may limit creatine production in IUGR piglets. Creatine is crucial for energy metabolism and antioxidative responses in animal tissues, particularly skeletal muscle and brain [6,79,80], whereas GSH is a potent antioxidant in all cell types [81,82,83]. Due to high intramuscular concentrations of creatine and GSH (Table 7 and Table 8), muscle growth requires the accretion of large amounts of creatine [84,85,86,87] and GSH [88,89,90], as well as intracellular proteins. This is of enormous physiological significance, as the biochemical process of protein synthesis accounts for ~15% of whole-body energy expenditure in young growing mammals, including piglets [91]. Additionally, increased growth rates are concomitant with increases in cellular energy metabolism and the production of reactive oxygen species [91]. These oxidants must be removed through the actions of antioxidants (including GSH) to maintain a proper redox balance in the animal body. Consistent with this notion, increasing glycine supplementation from 0.2 to 0.8 g/kg BW/day dose-dependently decreased concentrations of TBARS in the plasma of sow-reared piglets (Table 6).

Unlike the effects of oral administration of arginine, the oral administration of up to 0.8 g glycine/kg BW/day to sow-reared IUGR piglets did not affect the circulating levels of cortisol, growth hormone, insulin, or IGF-I (Table 6). Similarly, the intravenous infusion of physiological doses of glycine (1.1 g/min for 20 min) to healthy young adults was reported not to influence the secretion or circulating level of cortisol [92]. In addition, neither the intravenous infusion of glycine (0.6 g over 30 min) to adult men [93] nor dietary glycine supplementation (0.3 g/kg BW) to mice via drinking water for 6 weeks [94] affected the secretion or circulating level of insulin. Moreover, single oral administration of glycine (57, 113, and 225 mg/100 g BW) to food-deprived (24 h) chicks had acute (2 h later) dose-dependent effects on reducing the concentrations of corticosterone in plasma without affecting those of insulin [95]. It is possible that the effect of glycine on hormonal secretion in vivo depends on nutritional state. Likewise, adding 0.5–1 mM glycine to culture medium failed to increase gene expression or release of IGF-I by hepatocytes [96]. Interestingly, an acute intravenous infusion of high amounts of glycine (4, 8, or 12 g over 30 min) dose-dependently increased concentrations of growth hormone in the plasma of healthy adult humans [97]. To date, physiological levels of glycine are not known to stimulate the secretion of growth hormone in pigs [98,99]. Based on the results of the present work, it is unlikely that the beneficial function of glycine supplementation to enhance the growth of IUGR pigs is mediated by an effect of either cortisol, insulin, growth hormone, or IGF-I.

Through cell signaling, MTOR is the master regulator of protein biosynthesis, cell growth, and cytoskeleton remodeling in animal tissues [100,101,102,103]. The development of skeletal muscle depends on the activation of the MTOR system that consists of MTOR complex 1 and MTOR complex 2. Both complexes are phosphorylated by an upstream protein kinase [100]. Upon being phosphorylated, the activated MTOR phosphorylates its two downstream target proteins: p70^S6K^ and 4E-BP1. This cell signaling cascade leads to the formation of the translationally active 80S ribosome to initiate protein synthesis in tissues (including skeletal muscle) [101]. Results of in vitro studies with cultured C2C12 muscle cells showed that physiological concentrations of glycine activate the MTOR cell signaling pathway to stimulate protein synthesis and cell growth [57,104,105]. Specifically, adding 0.25, 0.5, or 1.0 mM glycine (within the physiological range for concentrations of glycine in the plasma of mammals [91]) to glycine-free culture medium enhanced the MTOR signaling pathway in C2C12 cells [57,104,105] and intestinal epithelial cells [62,63] in a dose-dependent manner, thereby increasing protein synthesis and reducing proteolysis in both cell types. Oxidation of glycine to CO_2_ in muscle cells and the small intestine is very limited [106]. Additionally, based on the following lines of evidence, it is unlikely that amino acids other than glycine contribute to MTOR activation in the tissues (including skeletal muscle and small intestine) of glycine-supplemented piglets. First, the addition of serine (0.4–2 mM) to culture medium did not activate MTOR in muscle cells [107] or intestinal epithelial cells [108]. Second, oral administration of serine to adult humans increased concentrations of serine in plasma but failed to activate MTOR in their skeletal muscle [109]. Third, concentrations of extracellular serine at up to 1 mM did not affect MTOR signaling in normal or malignant cells [110]. Fourth, the concentrations of all amino acids except for glycine and serine in the plasma and tissues of IUGR piglets were affected by dietary glycine supplementation (Table 4). However, it is unknown whether GSH and creatine play a role in mediating the effect of glycine on MTOR activation through alleviating oxidative stress and enhancing ATP regeneration for energy metabolism in tissues. Further research is warranted to test this hypothesis. Nonetheless, to our knowledge, this is the first report of an important role of dietary glycine in increasing MTOR cell signaling and protein synthesis in the skeletal muscle of animals. How glycine activates MTOR remains to be elucidated. Glycine may directly bind to MTOR, leading to a conformational change in the protein kinase. In an analogous manner, glycine has been reported to bind glycine receptors in the brain and spinal cord to modulate neuronal activity in the central nervous system [111]. The novel hypothesis that glycine allosterically activates MTOR in cells should be tested in future investigations. Additionally, research on mRNA levels of MTOR, 4E-BP1 and p70^S6K^ (which were not measured in the present work) is warranted to better understand how dietary glycine regulates gene expression in pig tissues. Given the significant problem of IUGR in livestock and other mammals [112,113,114,115,116,117], our present findings have important implications for enhancing the growth of preweaning IUGR piglets as previously reported for sow-reared NBW piglets [118], postweaning IUGR piglets [40], and postweaning NBW piglets [17,119,120,121,122,123].

## 5. Conclusions

**Figure 4:**
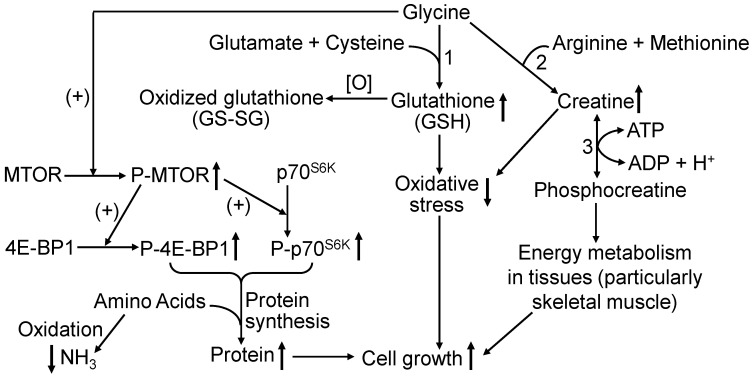
Biochemical mechanisms whereby glycine promotes the growth of sow-reared IUGR piglets. Glycine stimulates the phosphorylation of the mechanistic target of rapamycin (MTOR), which subsequently phosphorylates its two downstream proteins, eukaryotic initiation factor 4E binding protein 1 (4E-BP1) and ribosomal protein S6 kinase beta-1 (p70^S6K^), resulting in the initiation of protein synthesis in cells. Glycine (the most abundant amino acid in the body) and other proteinogenic amino acids serve as the building blocks of proteins. An increase in the use of amino acids for protein synthesis reduces their availability for oxidation and NH_3_ production. Notably, the process of protein synthesis requires a large amount of energy (representing ~15% of whole-body energy expenditure in young growing pigs) and is favored by both creatine and glutathione (for antioxidative and ATP-regenerating reactions). The formation of glutathione (Step 1) requires glycine, glutamate, and cysteine as substrates, whereas the synthesis of creatine (Step 2) involves the interorgan metabolism of glycine, arginine, and methionine (mainly the kidneys, pancreas, liver, and skeletal muscle). Through its conversion to phosphocreatine by creatine kinase (Step 3), creatine plays an important role in intracellular energy metabolism. Thus, glycine enhances the growth of piglets via activating the MTOR cell signaling, preventing oxidative stress, and generating sufficient amounts of ATP. ↑, increase; ↓, decrease; (+), activation.

Oral administration of glycine (0.2, 0.4 and 0.8 g/kg BW per day) to sow-reared IUGR piglets enhanced the availabilities of glycine, GSH, and creatine in plasma and tissues, as well as growth performance and lean tissue gain, while reducing the concentrations of ammonia, urea, and TBARS in plasma. Glycine supplementation did not affect the circulating levels of cortisol, insulin, growth hormone, or IGF-I, but activated the MTOR cell signaling pathway to promote protein synthesis and accretion in skeletal muscle and other tissues. Piglets tolerated glycine with no adverse effects at all from the supplemental doses. These results highlight a functional role for glycine in nutrition and metabolism (Figure 4) and support the view that the endogenous synthesis of glycine in piglets is insufficient for their maximal growth. Clearly, glycine is a conditionally essential amino acid that benefits the growth and development of piglets, especially IUGR piglets, during the preweaning period of their life.

## Figures and Tables

**Table 1:** Composition of the diet for lactating sows (as-fed basis).

Item	% (as-Fed Basis) ^1^
Ingredient	
Corn grain	57.50
Soybean meal, 44.5% crude protein	27.00
Cornstarch	2.0
Sugarcane molasses	3.85
Potassium chloride	0.10
Salt	0.35
Vitamin–mineral premix ^2^	3.00
Vegetable oil	3.00
Dicalcium phosphate	2.50
Limestone	0.70
Chemical composition	
Dry matter, %	90.0
Metabolizable energy, Mcal/kg	3.32
Crude protein, %	17.5
Calcium, %	1.04
Available phosphorus, %	0.54
Total phosphorus, %	0.79

^1^ This lactation diet was provided to sows ad libitum from the day of farrowing. ^2^ The vitamin premix provided the following per kg of complete diet (as-fed basis): 46.7 mg of Mn as manganous oxide; 75 mg of Fe as iron sulfate; 103.8 mg of Zn as zinc oxide; 9.5 mg of Cu as copper sulfate; 0.72 mg of I as ethylenediamine dihydroiodide; 0.23 mg of Se as sodium selenite; 7556 IU of vitamin A as vitamin A acetate; 825 IU of vitamin D_3_; 61.9 IU of vitamin E; 4.4 IU of vitamin K as menadione sodium bisulfate; 54.9 µg of vitamin B_12_; 13.7 mg of riboflavin; 43.9 mg of D-pantothenic acid as calcium pantothenate; 54.9 mg of niacin; and 1650 mg of choline as choline chloride.

**Table 2:** Effects of oral administration of glycine on the growth of sow-reared IUGR piglets ^1^.

Age	Oral Administration of Glycine (g/kg Body Weight/day)			
(days)	0	0.2	0.4	0.8
	Body weight (kg)			
0	0.86 ± 0.07	0.88 ± 0.02	0.85 ± 0.04	0.88 ± 0.04
7	1.63 ± 0.08 ^b^	1.88 ± 0.06 ^a^	2.07 ± 0.05 ^a^	2.01 ± 0.08 ^a^
14	2.30 ± 0.12 ^c^	2.76 ± 0.08 ^b^	3.16 ± 0.14 ^a^	3.07 ± 0.07 ^a^
	Weight gain (kg)			
Days 7–0	0.77 ± 0.04 ^c^	1.00 ± 0.05 ^b^	1.22 ± 0.02 ^a^	1.14 ± 0.05 ^a,b^
Days 14–7	0.67 ± 0.04 ^c^	0.88 ± 0.04 ^b^	1.10 ± 0.09 ^a^	1.06 ± 0.06 ^a^
Days 14–0	1.44 ± 0.08 ^c^	1.88 ± 0.06 ^b^	2.31 ± 0.10 ^a^	2.20 ± 0.04 ^a^
	*p*-values			
	Glycine	Age	Glycine × Age	
Body weight	<0.001	<0.001	0.862	
Weight gain	<0.001	<0.001	0.735	

^1^ Values are means ± SEM, n = 10. Piglets with intrauterine growth restriction (IUGR) received oral administration of either 0 (control), 0.1, 0.2 or 0.4 g glycine/kg body weight twice daily between 0 and 14 days of age. Alanine was used as the isonitrogenous control. Body weights of 10 surviving piglets in each treatment group were recorded at 0, 7 and 14 days of age. Data were analyzed by two-way ANOVA and the Student–Newman–Keuls multiple comparison test. ^a–c^: Within a row, means not sharing the same superscript letter differ (*p* < 0.05).

**Table 3:** Effects of oral administration of glycine on weights of tissues from sow-reared IUGR piglets ^1^.

Tissue	Oral Administration of Glycine (g/kg Body Weight/day)				
	0	0.2	0.4	0.8	*p*-Value
	Absolute Tissue Weight (g)				
LLM ^2^	7.36 ± 0.22 ^c^	9.02 ± 0.19 ^b^	10.5 ± 0.28 ^a^	10.1 ± 0.34 ^a^	<0.001
GM	32.9 ± 0.73 ^c^	40.1 ± 1.5 ^b^	46.9 ± 0.76 ^a^	45.7 ± 1.2 ^a^	<0.001
SI	84.0 ± 1.8 ^c^	102.6 ± 2.5 ^b^	116.4 ± 3.7 ^a^	112.9 ± 4.3 ^a^	<0.001
Liver	70.6 ± 1.9 ^c^	85.1 ± 1.5 ^b^	97.9 ± 2.8 ^a^	94.6 ± 4.0 ^a^	<0.001
Kidneys	16.6 ± 0.40 ^c^	20.1 ± 0.56 ^b^	23.2 ± 0.69 ^a^	22.6 ± 0.63 ^a^	<0.001
Pancreas	3.20 ± 0.12 ^c^	3.91 ± 0.09 ^b^	4.67 ± 0.29 ^a^	4.42 ± 0.15 ^a^	<0.001
Stomach	10.7 ± 0.43 ^c^	13.2 ± 0.35 ^b^	15.3 ± 0.40 ^a^	14.6 ± 0.21 ^a^	<0.001
Heart	13.5 ± 0.39 ^c^	16.0 ± 0.61 ^b^	18.5 ± 0.57 ^a^	18.1 ± 0.45 ^a^	<0.001
	Percentage of Body Weight (%)				
LLM	0.33 ± 0.02	0.32 ± 0.01	0.33 ± 0.01	0.33 ± 0.02	0.996
GM	1.47 ± 0.08	1.44 ± 0.02	1.48 ± 0.04	1.49 ± 0.06	0.925
SI	3.76 ± 0.20	3.70 ± 0.08	3.65 ± 0.05	3.66 ± 0.13	0.928
Liver	3.16 ± 0.15	3.07 ± 0.06	3.08 ± 0.05	3.06 ± 0.15	0.946
Kidneys	0.74 ± 0.04	0.72 ± 0.02	0.73 ± 0.02	0.73 ± 0.02	0.962
Pancreas	0.14 ± 0.01	0.14 ± 0.01	0.15 ± 0.01	0.14 ± 0.01	0.861
Stomach	0.48 ± 0.03	0.47 ± 0.01	0.48 ± 0.02	0.47 ± 0.01	0.965
Heart	0.60 ± 0.03	0.57 ± 0.02	0.58 ± 0.03	0.59 ± 0.02	0.856

^1^ Values are means ± SEM, n = 6. Piglets with intrauterine growth restriction (IUGR) received oral administration of either 0 (control), 0.1, 0.2 or 0.4 g glycine/kg body weight twice daily between 0 and 14 days of age. Alanine was used as the isonitrogenous control. At 14 days of age, after blood sampling, 6 pigs were selected randomly from each treatment group for euthanasia and then tissue collection. Data were analyzed by one-way ANOVA and the Student–Newman–Keuls multiple comparison test. ^2^ Obtained from the last five ribs. ^a–c^: Within a row, means not sharing the same superscript letter differ (*p* < 0.05). GM, gastrocnemius muscle; LLM, longissimus lumborum muscle; SI, small intestine.

**Table 4:** Effects of dietary supplementation with glycine on concentrations of free amino acids in the plasma of sow-reared IUGR piglets ^1^.

Amino Acid	Oral Administration of Glycine (g/kg Body Weight/day)				
	0	0.2	0.4	0.8	*p*-Value
	nmol/mL				
Alanine	903 ± 9.6 ^a^	627 ± 6.1 ^b^	613 ± 8.5 ^b^	615 ± 7.6 ^b^	<0.001
β-Alanine	7.3 ± 1.1	7.1 ± 0.8	6.9 ± 0.5	7.2 ± 0.7	0.987
Arginine	120 ± 9.2	122 ± 7.2	127 ± 5.7	125 ± 8.9	0.925
Aspartate	14.1 ± 1.6	15.3 ± 1.7	14.7 ± 1.7	16.0 ± 2.5	0.908
Asparagine	82.2 ± 6.1	83.4 ± 6.3	79.5 ± 9.9	75.2 ± 5.7	0.857
Citrulline	52.8 ± 1.6	54.4 ± 2.8	51.6 ± 3.1	53.6 ± 4.6	0.936
Cysteine	182 ± 10	177 ± 12	180 ± 13	186 ± 11	0.955
Glutamate	189 ± 12 ^a^	161 ± 11 ^a,b^	144 ± 8.9 ^b^	137 ± 9.6 ^b^	0.010
Glutamine	654 ± 18 ^a^	547 ± 19 ^b^	478 ± 21 ^c^	455 ± 4.9 ^c^	<0.001
Glycine	586 ± 10 ^a^	890 ± 17 ^b^	1134 ± 19 ^c^	1382 ± 22 ^d^	<0.001
Histidine	67.2 ± 4.3	65.4 ± 3.4	64.6 ± 5.3	67.9 ± 7.2	0.967
4-Hydroxyproline	78.0 ± 2.7	75.7 ± 4.2	76.2 ± 3.2	77.3 ± 3.5	0.964
Isoleucine	106 ± 5.8	112 ± 4.7	107 ± 9.4	110 ± 8.2	0.933
Leucine	151 ± 6.8	155 ± 4.0	153 ± 6.5	148 ± 8.4	0.893
Lysine	136 ± 11	141 ± 9.4	149 ± 11	153 ± 9.0	0.639
Methionine	60.1 ± 4.1	62.5 ± 5.3	67.5 ± 7.7	66.9 ± 4.9	0.759
Ornithine	57.0 ± 4.2	59.5 ± 3.1	57.5 ± 6.1	55.6 ± 3.2	0.935
Phenylalanine	71.5 ± 5.0	64.0 ± 3.9	71.0 ± 8.7	74.3 ± 5.8	0.678
Proline	287 ± 17	291 ± 15	283 ± 16	280 ± 15	0.964
Serine	278 ± 13 ^b^	312 ± 8.6 ^a,b^	328 ± 12 ^a^	347 ± 6.8 ^a^	0.001
Taurine	124 ± 2.7	126 ± 11	129 ± 5.4	127 ± 3.8	0.959
Threonine	154 ± 7.7 ^a^	171 ± 6.1 ^a,b^	189 ± 11 ^b^	194 ± 11 ^b^	0.024
Tryptophan	76.0 ± 5.1	73.2 ± 2.9	72.2 ± 5.6	76.1 ± 4.1	0.902
Tyrosine	154 ± 7.2	149 ± 4.7	156 ± 9.1	157 ± 8.2	0.877
Valine	210 ± 9.1	211 ± 5.1	216 ± 6.3	218 ± 4.2	0.783

^1^ Values are means ± SEM, n = 6. Piglets with intrauterine growth restriction (IUGR) received oral administration of either 0 (control), 0.1, 0.2 or 0.4 g glycine/kg body weight twice daily between 0 and 14 days of age. Alanine was used as the isonitrogenous control. At 14 days of age, blood samples were obtained from the jugular vein of 6 pigs chosen at random in each treatment group (see Table 3). Plasma was analyzed for free amino acids. Data were analyzed by one-way ANOVA and the Student–Newman–Keuls multiple comparison test. ^a–d^: Within a row, means not sharing the same superscript letter differ (*p* < 0.05).

**Table 5:** Effects of dietary supplementation with glycine on concentrations of free glycine and serine in tissues of sow-reared IUGR piglets ^1^.

Amino Acid	Oral Administration of Glycine (g/kg Body Weight/day)				
	0	0.2	0.4	0.8	*p*-Value
	Longissimus lumborum muscle (nmol/g of wet tissue)				
Glycine	2108 ± 79 ^d^	2635 ± 64 ^c^	3221 ± 87 ^b^	3958 ± 96 ^a^	<0.001
Serine	2564 ± 69 ^c^	2893 ± 66 ^b^	3124 ± 79 ^a^	3181 ± 75 ^a^	<0.001
	Gastrocnemius muscle (nmol/g of wet tissue)				
Glycine	2264 ± 88 ^d^	2986 ± 92 ^c^	3706 ± 108 ^b^	4483 ± 112 ^a^	<0.001
Serine	2487 ± 74 ^c^	2793 ± 81 ^b^	3065 ± 91 ^a^	3124 ± 96 ^a^	<0.001
	Liver (nmol/g of wet tissue)				
Glycine	4413 ± 120 ^d^	5290 ± 109 ^c^	6193 ± 147 ^b^	7253 ± 178 ^a^	<0.001
Serine	1060 ± 60 ^c^	1187 ± 55 ^b,c^	1341 ± 69 ^a,b^	1404 ± 75 ^a^	0.006
	Jejunum (nmol/g of wet tissue)				
Glycine	2304 ± 98 ^d^	2806 ± 67 ^c^	3367 ± 80 ^b^	3975 ± 77 ^a^	<0.001
Serine	1296 ± 65 ^b^	1420 ± 60 ^a,b^	1581 ± 55 ^a^	1635 ± 74 ^a^	0.005
	Kidney (nmol/g of wet tissue)				
Glycine	7324 ± 112 ^d^	8430 ± 168 ^c^	9855 ± 367 ^b^	12,086 ± 574 ^a^	<0.001
Serine	869 ± 32 ^c^	923 ± 36 ^b,c^	1038 ± 41 ^a,b^	1097 ± 48 ^a^	0.004

^1^ Values are means ± SEM, n = 6. Piglets with intrauterine growth restriction (IUGR) received oral administration of either 0 (control), 0.1, 0.2 or 0.4 g glycine/kg body weight twice daily between 0 and 14 days of age. Alanine was used as the isonitrogenous control. At 14 days of age, tissues were obtained from six pigs in each treatment group (see Table 3). Each tissue sample was analyzed for free amino acids. Data were analyzed by one-way ANOVA and the Student–Newman–Keuls multiple comparison test. ^a–d^: Within a row, means not sharing the same superscript letter differ (*p* < 0.05).

**Table 6:** Concentrations of glucose, nitrogenous metabolites, TBARS, lipids, and hormones in the plasma or serum of sow-reared IUGR piglets receiving oral administration of glycine ^1^.

Glucose or Hormone Concentrations	Oral Administration of Glycine (g/kg BW/day)				*p*-Value
	0	0.2	0.4	0.8	
Glucose in plasma, mM	5.73 ± 0.17	5.64 ± 0.23	5.60 ± 0.20	5.84 ± 0.12	0.802
Ammonia in plasma, μM	141 ± 5.7 ^a^	124 ± 5.5 ^b^	106 ± 5.1 ^c^	104 ± 5.3 ^c^	<0.001
Urea in plasma, mM	2.18 ± 0.09 ^a^	1.96 ± 0.07 ^b^	1.70 ± 0.06 ^c^	1.73 ± 0.06 ^c^	<0.001
TBARS in plasma, μM	5.26 ± 0.23 ^a^	4.68 ± 0.21 ^b^	4.07 ± 0.16 ^c^	3.52 ± 0.11 ^d^	<0.001
Free fatty acids in plasma, μM	253 ± 16	260 ± 20	248 ± 14	272 ± 23	0.815
Triacylglycerols in plasma, μM	793 ± 48	812 ± 65	772 ± 46	788 ± 51	0.962
Total cholesterol in plasma, mM	2.03 ± 0.10	1.94 ± 0.08	2.07 ± 0.15	1.87 ± 0.12	0.618
Cortisol in plasma, nM	64.7 ± 3.1	63.2 ± 2.8	61.9 ± 3.4	62.6 ± 3.9	0.942
Insulin in serum, pM	60.9 ± 3.4	62.8 ± 3.0	61.2 ± 3.7	63.4 ± 2.7	0.933
Growth hormone in serum, pM	359 ± 16	376 ± 18	371 ± 13	365 ± 21	0.907
IGF-I in serum, µg/L	31.5 ± 1.4	30.9 ± 2.2	32.1 ± 1.8	33.0 ± 2.4	0.893

^1^ Values are means ± SEM, n = 6. Piglets with intrauterine growth restriction (IUGR) received oral administration of 0 (control), 0.2, 0.4 or 0.8 g glycine/kg body weight (BW) per day between 0 and 14 days of age. L-Alanine was used as the isonitrogenous control. Piglets were nursed by sows at will. At 14 days of age, blood samples were obtained from the jugular vein of piglets in each treatment group. Plasma was analyzed for thiobarbituric acid reactive substances (TBARS), glucose, lipids, and cortisol, whereas serum was analyzed for insulin, growth hormone, and insulin-like growth factor-I (IGF-I). ^a–d^: Within a row, means not sharing the same superscript letter differ (*p* < 0.05).

**Table 7:** Effects of dietary supplementation with glycine on concentrations of creatine and related substances in the plasma and tissues of sow-reared IUGR piglets ^1^.

Variable	Oral Administration of Glycine (g/kg Body Weight/day)				
	0	0.2	0.4	0.8	*p*-Value
	Plasma (nmol/mL)				
Guanidinoacetate	39.4 ± 1.0 ^c^	45.0 ± 1.5 ^b^	50.9 ± 1.8 ^a^	52.4 ± 2.3 ^a^	<0.001
Creatine	252 ± 8.3 ^c^	295 ± 8.8 ^b^	327 ± 11 ^a^	336 ± 13 ^a^	<0.001
Phosphocreatine (PCr)	ND	ND	ND	ND	---
Creatine + PCr	252 ± 8.3 ^c^	295 ± 8.8 ^b^	327 ± 11 ^a^	336 ± 13 ^a^	<0.001
Creatinine	38.1 ± 1.5	39.0 ± 1.9	41.8 ± 2.3	42.5 ± 2.6	0.408
	Longissimus lumborum muscle (μmol/g of wet tissue)				
Guanidinoacetate	0.17 ± 0.01	0.18 ± 0.02	0.18 ± 0.02	0.19 ± 0.02	0.892
Creatine	12.0 ± 0.45 ^c^	13.9 ± 0.57 ^b^	15.6 ± 0.51 ^a^	16.1 ± 0.63 ^a^	<0.001
Phosphocreatine (PCr)	19.5 ± 0.62 ^c^	22.4 ± 0.71 ^b^	24.7 ± 0.89 ^a^	25.6 ± 0.75 ^a^	0.001
Creatine + PCr	31.5 ± 1.1 ^c^	36.3 ± 1.2 ^b^	40.3 ± 1.4 ^a^	41.7 ± 1.4 ^a^	<0.001
Creatinine	1.10 ± 0.06	1.15 ± 0.08	1.24 ± 0.07	1.28 ± 0.06	0.255
	Gastrocnemius muscle (μmol/g of wet tissue)				
Guanidinoacetate	0.13 ± 0.01	0.14 ± 0.01	0.15 ± 0.01	0.16 ± 0.02	0.434
Creatine	11.2 ± 0.41 ^c^	13.0 ± 0.49 ^b^	14.8 ± 0.66 ^a^	15.3 ± 0.58 ^a^	0.001
Phosphocreatine (PCr)	18.3 ± 0.55 ^c^	20.8 ± 0.68 ^b^	23.9 ± 0.78 ^a^	24.4 ± 0.92 ^a^	<0.001
Creatine + PCr	29.5 ± 0.70 ^c^	33.7 ± 1.1 ^b^	38.7 ± 1.2 ^a^	39.7 ± 1.2 ^a^	<0.001
Creatinine	0.91 ± 0.03	0.93 ± 0.04	0.95 ± 0.05	0.97 ± 0.05	0.786
	Liver (μmol/g of wet tissue)				
Guanidinoacetate	0.21 ± 0.02	0.23 ± 0.02	0.24 ± 0.02	0.25 ± 0.02	0.547
Creatine	1.93 ± 0.07 ^c^	2.24 ± 0.09 ^b^	2.52 ± 0.11 ^a^	2.60 ± 0.10 ^a^	<0.001
Phosphocreatine (PCr)	0.34 ± 0.02	0.36 ± 0.02	0.39 ± 0.03	0.41 ± 0.02	0.260
Creatine + PCr	2.27 ± 0.06 ^c^	2.60 ± 0.11 ^b^	2.92 ± 0.12 ^a^	3.01 ± 0.10 ^a^	0.001
Creatinine	0.11 ± 0.01	0.12 ± 0.01	0.13 ± 0.02	0.13 ± 0.01	0.671
	Jejunum (μmol/g of wet tissue)				
Guanidinoacetate	0.051 ± 0.003	0.053 ± 0.003	0.055 ± 0.004	0.058 ± 0.004	0.558
Creatine	3.43 ± 0.10 ^c^	3.88 ± 0.14 ^b^	4.50 ± 0.16 ^a^	4.61 ± 0.19 ^a^	0.001
Phosphocreatine (PCr)	3.91 ± 0.12 ^c^	4.46 ± 0.17 ^b^	5.09 ± 0.21 ^a^	5.17 ± 0.18 ^a^	0.001
Creatine + PCr	7.34 ± 0.21 ^c^	8.34 ± 0.31 ^b^	9.59 ± 0.34 ^a^	9.77 ± 0.37 ^a^	0.001
Creatinine	0.13 ± 0.01	0.14 ± 0.01	0.14 ± 0.01	0.16 ± 0.02	0.459
	Kidney (μmol/g of wet tissue)				
Guanidinoacetate	0.68 ± 0.02 ^c^	0.78 ± 0.03 ^b^	0.89 ± 0.03 ^a^	0.91 ± 0.04 ^a^	0.001
Creatine	1.65 ± 0.06 ^c^	1.92 ± 0.07 ^b^	2.18 ± 0.09 ^a^	2.24 ± 0.11 ^a^	<0.001
Phosphocreatine (PCr)	1.16 ± 0.05 ^c^	1.34 ± 0.05 ^b^	1.57 ± 0.07 ^a^	1.64 ± 0.06 ^a^	<0.001
Creatine + PCr	2.81 ± 0.09 ^c^	3.26 ± 0.12 ^b^	3.75 ± 0.16 ^a^	3.88 ± 0.16 ^a^	<0.001
Creatinine	0.34 ± 0.02 ^c^	0.41 ± 0.02 ^b^	0.49 ± 0.03 ^a^	0.51 ± 0.02 ^a^	0.001

^1^ Values are means ± SEM, n = 6. Piglets with intrauterine growth restriction (IUGR) received oral administration of either 0 (control), 0.1, 0.2 or 0.4 g glycine/kg body weight twice daily between 0 and 14 days of age. Alanine was used as the isonitrogenous control. At 14 days of age, blood and tissues were obtained from 6 pigs in each treatment group (see Table 3). Plasma and tissues were analyzed for creatine and related substances. Data were analyzed by one-way ANOVA and the Student–Newman–Keuls multiple comparison test. ^a–c^: Within a row, means not sharing the same superscript letter differ (*p* < 0.05). ND, not detected.

**Table 8:** Effects of dietary supplementation with glycine on concentrations of reduced glutathione (GSH) and oxidized glutathione (GSSG) in the plasma and tissues of sow-reared IUGR piglets ^1^.

Variable	Oral Administration of Glycine (g/kg Body Weight/day)					
	0	0.2	0.4	0.8	*p*-Value	
	Plasma					
GSH, nmol/mL	4.58 ± 0.13 ^d^	5.18 ± 0.16 ^c^	5.92 ± 0.20 ^b^	6.68 ± 0.23 ^a^	<0.001	
GSSG, nmol/mL	0.768 ± 0.036 ^a^	0.723 ± 0.031 ^a,b^	0.685 ± 0.032 ^a,b^	0.637 ± 0.026 ^b^	0.045	
GSSG/GSH, nmol/nmol	0.169 ± 0.011 ^a^	0.140 ± 0.007 ^b^	0.116 ± 0.006 ^c^	0.096 ± 0.006 ^c^	<0.001	
	Longissimus lumborum muscle					
GSH, μmol/g of wet tissue	0.679 ± 0.031 ^d^	0.773 ± 0.034 ^c,d^	0.895 ± 0.040 ^b^	1.02 ± 0.05 ^a^	<0.001	
GSSG, μmol/g of wet tissue	0.118 ± 0.008 ^a^	0.106 ± 0.007 ^a,b^	0.095 ± 0.007 ^a,b^	0.089 ± 0.006 ^b^	0.041	
GSSG/GSH, μmol/μmol	0.173 ± 0.005 ^a^	0.138 ± 0.006 ^b^	0.105 ± 0.004 ^c^	0.088 ± 0.005 ^d^	<0.001	
	Gastrocnemius muscle					
GSH, μmol/g of wet tissue	0.818 ± 0.033 ^d^	0.902 ± 0.037 ^c,d^	1.04 ± 0.05 ^b^	1.18 ± 0.06 ^a^	<0.001	
GSSG, μmol/g of wet tissue	0.140 ± 0.009 ^a^	0.128 ± 0.008 ^a,b^	0.117 ± 0.007 ^a,b^	0.108 ± 0.006 ^b^	0.039	
GSSG/GSH, μmol/μmol	0.171 ± 0.007 ^a^	0.142 ± 0.006 ^b^	0.113 ± 0.009 ^c^	0.092 ± 0.003 ^d^	<0.001	
	Liver					
GSH, μmol/g of wet tissue	4.19 ± 0.14 ^d^	4.78 ± 0.18 ^c^	5.40 ± 0.20 ^b^	6.15 ± 0.23 ^a^	<0.001	
GSSG, μmol/g of wet tissue	0.282 ± 0.015 ^a^	0.252 ± 0.014 ^a,b^	0.237 ± 0.012 ^a,b^	0.227 ± 0.010 ^b^	0.032	
GSSG/GSH, μmol/μmol	0.067 ± 0.003 ^a^	0.053 ± 0.003 ^b^	0.044 ± 0.002 ^c^	0.037 ± 0.001 ^d^	<0.001	
	Jejunum					
GSH, μmol/g of wet tissue	1.04 ± 0.08 ^d^	1.28 ± 0.07 ^c^	1.53 ± 0.06 ^b^	1.78 ± 0.09 ^a^	<0.001	
GSSG, μmol/g of wet tissue	0.163 ± 0.008 ^a^	0.155 ± 0.006 ^a,b^	0.147 ± 0.008 ^a,b^	0.133 ± 0.006 ^b^	0.042	
GSSG/GSH, μmol/μmol	0.161 ± 0.011 ^a^	0.122 ± 0.006 ^b^	0.096 ± 0.004 ^c^	0.075 ± 0.003 ^d^	<0.001	
	Kidney					
GSH, μmol/g of wet tissue	0.732 ± 0.020 ^c^	0.798 ± 0.026 ^b,c^	0.881 ± 0.037 ^a,b^	0.962 ± 0.043 ^a^	<0.001	
GSSG, μmol/g of wet tissue	0.056 ± 0.003 ^a^	0.051 ± 0.003 ^a,b^	0.046 ± 0.003 ^a,b^	0.044 ± 0.003 ^b^	0.025	
GSSG/GSH, μmol/μmol	0.076 ± 0.003 ^a^	0.063 ± 0.002 ^b^	0.053 ± 0.002 ^c^	0.046 ± 0.001 ^d^	<0.001	

^1^ Values are means ± SEM, n = 6. Piglets with intrauterine growth restriction (IUGR) received oral administration of either 0 (control), 0.1, 0.2, or 0.4 g glycine/kg body weight twice daily between 0 and 14 days of age. Alanine was used as the isonitrogenous control. At 14 days of age, blood and tissues were obtained from 6 pigs in each treatment group (see Table 3). Plasma and tissues were analyzed for creatine and related substances. Data were analyzed by one-way ANOVA and the Student–Newman–Keuls multiple comparison test. ^a–d^: Within a row, means not sharing the same superscript letter differ (*p* < 0.05).

**Table 9:** Effects of dietary supplementation with glycine on protein synthesis in tissues of sow-reared IUGR piglets ^1^.

Variable	Oral Administration of Glycine (g/kg Body Weight/day)		
	0	0.4	*p*-Value
	Longissimus lumborum muscle ^2^		
Fractional rate of protein synthesis, %/day	14.1 ± 0.62	16.3 ± 0.71	0.035
Amount of protein per dissected tissue, g	1.39 ± 0.05	1.82 ± 0.09	<0.001
Absolute rate of protein synthesis, g/day	0.20 ± 0.013	0.30 ± 0.020	0.001
	Gastrocnemius muscle		
Fractional rate of protein synthesis, %/day	15.3 ± 0.59	17.6 ± 0.74	0.029
Amount of protein per tissue, g	6.28 ± 0.23	8.33 ± 0.44	0.001
Absolute rate of protein synthesis, g/day	0.96 ± 0.05	1.47 ± 0.09	<0.001
	Liver		
Fractional rate of protein synthesis, %/day	80.6 ± 3.2	94.5 ± 4.1	0.018
Amount of protein per tissue, g	11.2 ± 0.43	14.6 ± 0.45	<0.001
Absolute rate of protein synthesis, g/day	9.09 ± 0.61	13.8 ± 0.82	<0.001
	Jejunum		
Fractional rate of protein synthesis, %/day	57.2 ± 2.4	67.5 ± 2.7	0.013
Amount of protein per tissue, g	9.71 ± 0.49	12.6 ± 0.79	0.008
Absolute rate of protein synthesis, g/day	5.54 ± 0.30	8.64 ± 0.83	0.003
	Kidneys		
Fractional rate of protein synthesis, %/day	36.2 ± 1.5	42.0 ± 2.1	0.041
Amount of protein in two kidneys, g	1.93 ± 0.11	2.59 ± 0.14	0.002
Absolute rate of protein synthesis, g/day	0.70 ± 0.05	1.08 ± 0.07	<0.001

^1^ Values are means ± SEM, n = 8. Piglets with intrauterine growth restriction (IUGR) received oral administration of either 0 (control) or 0.4 g glycine/kg body weight twice daily between 0 and 14 days of age. Alanine was used as the isonitrogenous control. At 14 days of age, rates of protein synthesis in piglet tissues were measured using the [^3^H]phenylalanine flooding dose technique. Data were analyzed by the unpaired *t*-test. ^2^ Obtained from the last five ribs.

## Data Availability

All data are contained within this article.

## Supplementary Materials

The following supporting information can be downloaded at: https://www.mdpi.com/article/10.3390/ani15131855/s1, Table S1. Effects of dietary supplementation with glycine on concentrations of free amino acids in the longissimus lumborum muscle of IUGR piglets ^1^. Table S2. Effects of dietary supplementation with glycine on concentrations of free amino acids in the gastrocnemius muscle of IUGR piglets ^1^. Table S3. Effects of dietary supplementation with glycine on concentrations of free amino acids in the liver of IUGR piglets ^1^. Table S4. Effects of dietary supplementation with glycine on concentrations of free amino acids in the jejunum of IUGR piglets ^1^. Table S5. Effects of dietary supplementation with glycine on concentrations of free amino acids in the kidneys of IUGR piglets ^1^. Figure S1. Littermate piglets with intrauterine growth restriction (IUGR) and normal birth weight (NBW) at 14 days of age. At birth, runt piglets may weigh only one half or even one third as much as their largest littermates.
