# Supplementary material for: Glycine Supplementation Enhances the Growth of Sow-Reared Piglets with Intrauterine Growth Restriction
Source: Animals (Basel). 2025 Jun 23;15(13):1855. doi: 10.3390/ani15131855 (PMC12249007; doi:10.3390/ani15131855)
Supplement: Supplementary file 1 [file animals-15-01855-s001.zip › animals-3680801-supplementary.pdf]

animals

MDPI

Supplementary Table S1. Effects of dietary supplementation with glycine on concentrations of free amino acids in the longissimus lumborum muscle of IUGR piglets[1].

Amino Acid	Oral Administration of Glycine (g/kg body weight/day)				
	0	0.2	0.4	0.8	*p*-value
	nmol/g of wet tissue				
Alanine	4529 ± 261	4485 ± 198	4437 ± 235	4331 ± 247	0.941
β-Alanine	703 ± 22	708 ± 31	729 ± 28	722 ± 36	0.899
Arginine	140 ± 8.3	144 ± 6.1	150 ± 7.9	142 ± 7.4	0.801
Aspartate	1016 ± 79	1078 ± 88	1034 ± 97	1129 ± 83	0.798
Asparagine	532 ± 36	514 ± 20	535 ± 26	530 ± 24	0.947
Citrulline	109 ± 4.6	106 ± 5.3	103 ± 6.3	112 ± 7.6	0.748
Cysteine	128 ± 6.8	122 ± 6.1	120 ± 9.2	125 ± 8.7	0.895
Glutamate	3549 ± 116	3468 ± 133	3401 ± 167	3356 ± 147	0.790
Glutamine	4086 ± 157	3958 ± 178	3849 ± 204	3802 ± 164	0.679
Glycine	2108 ± 79[d]	2635 ± 64[c]	3221 ± 87[b]	3958 ± 96[a]	< 0.001
Histidine	284 ± 12	276 ± 10	271 ± 8.8	266 ± 14	0.716
4-Hydroxyproline	51.3 ± 3.8	53.5 ± 2.3	52.0 ± 2.7	52.8 ± 2.4	0.952
Isoleucine	294 ± 14	287 ± 11	282 ± 8.4	292 ± 9.2	0.864
Leucine	481 ± 20	486 ± 16	494 ± 27	507 ± 22	0.843
Lysine	558 ± 23	542 ± 20	553 ± 29	546 ± 17	0.960
Methionine	154 ± 5.8	150 ± 8.4	155 ± 7.1	151 ± 6.0	0.948
Ornithine	74.3 ± 4.8	75.8 ± 4.3	73.8 ± 3.6	76.1 ± 2.8	0.970
Phenylalanine	355 ± 18	351 ± 22	360 ± 13	347 ± 16	0.959
Proline	2065 ± 69	1982 ± 106	2105 ± 84	2072 ± 92	0.624
Serine	2564 ± 69[c]	2893 ± 66[b]	3124 ± 79[a]	3181 ± 75[a]	< 0.001
Taurine	6019 ± 196	6084 ± 168	6164 ± 227	6031 ± 183	0.950
Threonine	1115 ± 84	1168 ± 92	1217 ± 89	1241 ± 97	0.769
Tryptophan	90.6 ± 3.8	88.7 ± 5.3	87.4 ± 4.2	92.2 ± 6.1	0.907
Tyrosine	373 ± 11	361 ± 9.3	369 ± 13	365 ± 16	0.916
Valine	635 ± 20	629 ± 31	624 ± 16	616 ± 28	0.955

[1] Values are means ± SEM, n = 6. Piglets with intrauterine growth restriction (IUGR) received oral administration of either 0 (control), 0.1, 0.2 or 0.4 g glycine/kg body weight twice daily between 0 and 14 days of age. Alanine was used as the isonitrogenous control. At 14 days of age, longissimus lumborum muscle was obtained from 6 pigs in each treatment group (see Table 3). The tissue was analyzed for free amino acids. Data were analyzed by one-way ANOVA and the Student-Newman-Keuls multiple comparison test. [a-d]: Within a row, means not sharing the same superscript letter differ ($p < 0.05$).

 https://doi.org/10.3390/ani15131855

Supplementary Table S2. Effects of dietary supplementation with glycine on concentrations of free amino acids in the gastrocnemius muscle of IUGR piglets[1].

**Amino Acid**	**Oral Administration of Glycine (g/kg body weight/day)**				
	**0**	**0.2**	**0.4**	**0.8**	***p*-value**
	nmol/g of wet tissue				
Alanine	4038 ± 220	3907 ± 236	3961 ± 186	3937 ± 165	0.972
β-Alanine	761 ± 23	735 ± 46	741 ± 43	729 ± 33	0.936
Arginine	158 ± 6.5	154 ± 7.5	152 ± 8.9	160 ± 8.1	0.882
Aspartate	1135 ± 73	1104 ± 60	1078 ± 77	1156 ± 65	0.863
Asparagine	585 ± 38	574 ± 23	549 ± 32	566 ± 35	0.883
Citrulline	102 ± 7.7	104 ± 5.0	107 ± 8.6	110 ± 7.3	0.873
Cysteine	119 ± 5.3	125 ± 7.8	116 ± 9.4	121 ± 6.6	0.855
Glutamate	3305 ± 118	3277 ± 103	3206 ± 153	3158 ± 141	0.849
Glutamine	5063 ± 125	4880 ± 142	4782 ± 161	4704 ± 132	0.331
Glycine	2264 ± 88[d]	2986 ± 92[c]	3706 ± 108[b]	4483 ± 112[a]	< 0.001
Histidine	273 ± 12	262 ± 9.4	269 ± 16	280 ± 8.8	0.754
4-Hydroxyproline	52.0 ± 1.9	51.2 ± 2.3	50.5 ± 3.0	52.3 ± 1.7	0.943
Isoleucine	289 ± 18	283 ± 14	292 ± 16	296 ± 21	0.960
Leucine	452 ± 23	441 ± 12	460 ± 26	448 ± 19	0.931
Lysine	551 ± 19	532 ± 28	536 ± 21	540 ± 24	0.945
Methionine	152 ± 8.7	148 ± 6.1	154 ± 6.8	146 ± 9.5	0.886
Ornithine	76.0 ± 4.5	77.3 ± 3.6	75.0 ± 4.1	78.3 ± 5.1	0.953
Phenylalanine	362 ± 13	355 ± 18	348 ± 15	351 ± 20	0.947
Proline	2147 ± 85	2106 ± 89	2208 ± 122	2093 ± 102	0.850
Serine	2487 ± 74[c]	2793 ± 81[b]	3065 ± 91[a]	3124 ± 96[a]	< 0.001
Taurine	9211 ± 325	9174 ± 373	9294 ± 319	9188 ± 416	0.995
Threonine	1178 ± 75	1219 ± 84	1278 ± 73	1326 ± 80	0.567
Tryptophan	87.8 ± 4.6	87.3 ± 3.9	85.1 ± 3.5	86.8 ± 4.3	0.969
Tyrosine	409 ± 21	391 ± 16	402 ± 19	395 ± 13	0.892
Valine	579 ± 28	561 ± 20	588 ± 24	574 ± 17	0.862

[1] Values are means ± SEM, n = 6. Piglets with intrauterine growth restriction (IUGR) received oral administration of either 0 (control), 0.1, 0.2 or 0.4 g glycine/kg body weight twice daily between 0 and 14 days of age. Alanine was used as the isonitrogenous control. At 14 days of age, gastrocnemius muscle was obtained from 6 pigs in each treatment group (see Table 3). The tissue was analyzed for free amino acids. Data were analyzed by one-way ANOVA and the Student-Newman-Keuls multiple comparison test. [a-d]: Within a row, means not sharing the same superscript letter differ ($p < 0.05$).

Supplementary Table S3. Effects of dietary supplementation with glycine on concentrations of free amino acids in the liver of IUGR piglets[1].

Amino Acid	Oral Administration of Glycine (g/kg body weight/day)				*p*-value
	0	0.2	0.4	0.8	
	nmol/g of wet tissue				
Alanine	4175 ± 251	4024 ± 240	4112 ± 276	3984 ± 208	0.945
β-Alanine	34.7 ± 2.2	33.2 ± 2.9	32.8 ± 1.8	34.0 ± 1.5	0.927
Arginine	70.8 ± 3.2	69.8 ± 2.4	69.3 ± 2.1	71.2 ± 2.8	0.954
Aspartate	1213 ± 89	1142 ± 77	1109 ± 68	1183 ± 83	0.806
Asparagine	542 ± 30	529 ± 21	517 ± 18	536 ± 25	0.891
Citrulline	32.7 ± 2.3	31.2 ± 1.7	31.8 ± 1.4	33.0 ± 2.1	0.904
Cysteine	242 ± 13	235 ± 16	228 ± 10	232 ± 18	0.919
Glutamate	4174 ± 183	4091 ± 203	4059 ± 254	4116 ± 211	0.984
Glutamine	3480 ± 190	3573 ± 147	3362 ± 202	3406 ± 162	0.844
Glycine	4413 ± 120[d]	5290 ± 109[c]	6193 ± 147[b]	7253 ± 178[a]	< 0.001
Histidine	705 ± 24	681 ± 36	673 ± 42	695 ± 31	0.911
4-Hydroxyproline	38.8 ± 2.0	37.2 ± 1.7	36.3 ± 1.5	37.5 ± 2.3	0.827
Isoleucine	356 ± 17	332 ± 13	351 ± 21	346 ± 24	0.832
Leucine	726 ± 34	713 ± 38	724 ± 27	733 ± 30	0.978
Lysine	512 ± 18	516 ± 24	508 ± 13	504 ± 28	0.982
Methionine	126 ± 7.4	120 ± 8.2	117 ± 9.3	124 ± 9.6	0.884
Ornithine	464 ± 26	442 ± 18	439 ± 34	457 ± 29	0.901
Phenylalanine	245 ± 16	237 ± 11	228 ± 19	233 ± 14	0.881
Proline	4159 ± 126	4094 ± 109	4118 ± 135	4052 ± 102	0.934
Serine	1060 ± 60[c]	1187 ± 55[b,c]	1341 ± 69[a,b]	1404 ± 75[a]	0.006
Taurine	9130 ± 203	9317 ± 286	9061 ± 237	9266 ± 212	0.860
Threonine	782 ± 29	771 ± 37	754 ± 26	766 ± 33	0.938
Tryptophan	107 ± 7.5	104 ± 6.4	102 ± 5.6	110 ± 7.1	0.844
Tyrosine	318 ± 11	310 ± 13	306 ± 19	322 ± 16	0.871
Valine	703 ± 30	698 ± 35	692 ± 32	710 ± 25	0.979

[1] Values are means ± SEM, n = 6. Piglets with intrauterine growth restriction (IUGR) received oral administration of either 0 (control), 0.1, 0.2 or 0.4 g glycine/kg body weight twice daily between 0 and 14 days of age. Alanine was used as the isonitrogenous control. At 14 days of age, the liver was obtained from 6 pigs in each treatment group (see Table 3). The tissue was analyzed for free amino acids. Data were analyzed by one-way ANOVA and the Student-Newman-Keuls multiple comparison test. [a-d]: Within a row, means not sharing the same superscript letter differ ($p < 0.05$).

Supplementary Table S4. Effects of dietary supplementation with glycine on concentrations of free amino acids in the jejunum of IUGR piglets[1].

**Amino Acid**	**Oral Administration of Glycine (g/kg body weight/day)**				
	**0**	**0.2**	**0.4**	**0.8**	***p*-value**
	nmol/g of wet tissue				
Alanine	3645 ± 105	3507 ± 128	3480 ± 93	3426 ± 114	0.558
β-Alanine	20.3 ± 1.1	21.2 ± 1.5	19.3 ± 1.2	20.7 ± 1.7	0.801
Arginine	853 ± 48	841 ± 36	846 ± 40	832 ± 57	0.990
Aspartate	1288 ± 67	1216 ± 98	1302 ± 74	1245 ± 88	0.876
Asparagine	551 ± 35	534 ± 26	540 ± 21	546 ± 29	0.976
Citrulline	191 ± 7.2	196 ± 10	202 ± 13	193 ± 8.7	0.851
Cysteine	201 ± 9.5	208 ± 9.1	191 ± 7.8	204 ± 13	0.672
Glutamate	4319 ± 154	4228 ± 146	4107 ± 119	4259 ± 170	0.782
Glutamine	1336 ± 92	1390 ± 83	1286 ± 67	1254 ± 60	0.620
Glycine	2304 ± 98[d]	2806 ± 67[c]	3367 ± 80[b]	3975 ± 77[a]	< 0.001
Histidine	648 ± 28	656 ± 32	641 ± 36	628 ± 26	0.929
4-Hydroxyproline	32.3 ± 2.1	30.2 ± 1.4	29.7 ± 1.7	30.8 ± 2.3	0.790
Isoleucine	402 ± 12	408 ± 16	394 ± 11	415 ± 19	0.782
Leucine	705 ± 19	702 ± 25	696 ± 21	711 ± 28	0.975
Lysine	729 ± 24	722 ± 14	714 ± 25	735 ± 20	0.907
Methionine	267 ± 17	271 ± 12	262 ± 19	264 ± 14	0.980
Ornithine	114 ± 8.0	110 ± 7.2	106 ± 8.5	121 ± 11	0.667
Phenylalanine	458 ± 11	439 ± 13	431 ± 17	447 ± 23	0.707
Proline	1367 ± 73	1297 ± 62	1263 ± 90	1316 ± 96	0.836
Serine	1296 ± 65[b]	1420 ± 60[a,b]	1581 ± 55[a]	1635 ± 74[a]	0.005
Taurine	9880 ± 461	9816 ± 385	9952 ± 407	9924 ± 344	0.996
Threonine	738 ± 40	723 ± 28	718 ± 33	727 ± 36	0.980
Tryptophan	126 ± 6.9	121 ± 7.4	117 ± 8.2	124 ± 9.1	0.865
Tyrosine	417 ± 19	405 ± 14	398 ± 12	410 ± 23	0.889
Valine	955 ± 61	963 ± 52	980 ± 70	971 ± 55	0.992

[1] Values are means ± SEM, n = 6. Piglets with intrauterine growth restriction (IUGR) received oral administration of either 0 (control), 0.1, 0.2 or 0.4 g glycine/kg body weight twice daily between 0 and 14 days of age. Alanine was used as the isonitrogenous control. At 14 days of age, the jejunum was obtained from 6 pigs in each treatment group (see Table 3). The intestinal luminal contents were removed and the intestinal lumen was washed with saline three times. The jejunal tissue was then analyzed for free amino acids. Data were analyzed by one-way ANOVA and the Student-Newman-Keuls multiple comparison test. [a-d]: Within a row, means not sharing the same superscript letter differ ($p < 0.05$).

Supplementary Table S5. Effects of dietary supplementation with glycine on concentrations of free amino acids in the kidneys of IUGR piglets[1].

**Amino Acid**	**Oral Administration of Glycine (g/kg body weight/day)**				
	**0**	**0.2**	**0.4**	**0.8**	***p*-value**
	nmol/g of wet tissue				
Alanine	3530 ± 178	3415 ± 201	3327 ± 157	3389 ± 149	0.866
β-Alanine	288 ± 20	276 ± 16	281 ± 12	293 ± 17	0.890
Arginine	271 ± 16	288 ± 21	265 ± 10	261 ± 15	0.651
Aspartate	1146 ± 69	1094 ± 82	1026 ± 57	1058 ± 76	0.676
Asparagine	1022 ± 74	1040 ± 85	1059 ± 68	1136 ± 109	0.793
Citrulline	64.3 ± 2.6	62.7 ± 3.6	63.2 ± 3.0	61.8 ± 3.3	0.953
Cysteine	331 ± 16	325 ± 13	346 ± 17	338 ± 11	0.760
Glutamate	6382 ± 186	6414 ± 228	6279 ± 175	6301 ± 191	0.955
Glutamine	335 ± 19	322 ± 14	319 ± 20	330 ± 14	0.905
Glycine	7324 ± 112[d]	8430 ± 168[c]	9855 ± 367[b]	12086 ± 574[a]	< 0.001
Histidine	378 ± 15	366 ± 17	386 ± 21	372 ± 13	0.854
4-Hydroxyproline	46.8 ± 2.4	45.3 ± 2.9	44.8 ± 2.6	46.2 ± 2.1	0.943
Isoleucine	272 ± 8.2	279 ± 10	266 ± 9.5	275 ± 9.0	0.786
Leucine	455 ± 11	449 ± 18	436 ± 15	452 ± 20	0.852
Lysine	421 ± 13	414 ± 16	410 ± 21	426 ± 18	0.915
Methionine	141 ± 7.6	145 ± 8.7	137 ± 6.6	132 ± 7.1	0.658
Ornithine	336 ± 15	348 ± 14	331 ± 18	352 ± 24	0.828
Phenylalanine	330 ± 10	318 ± 12	322 ± 15	335 ± 17	0.817
Proline	1571 ± 91	1622 ± 95	1531 ± 108	1594 ± 120	0.937
Serine	869 ± 32[c]	923 ± 36[b,c]	1038 ± 41[a,b]	1097 ± 48[a]	0.004
Taurine	5472 ± 202	5267 ± 151	5302 ± 166	5389 ± 137	0.833
Threonine	850 ± 30	893 ± 33	834 ± 23	872 ± 38	0.581
Tryptophan	119 ± 5.4	124 ± 8.1	115 ± 6.4	127 ± 8.4	0.655
Tyrosine	284 ± 12	272 ± 9.7	266 ± 14	278 ± 16	0.791
Valine	518 ± 19	504 ± 14	512 ± 17	523 ± 24	0.903

[1] Values are means ± SEM, n = 6. Piglets with intrauterine growth restriction (IUGR) received oral administration of either 0 (control), 0.1, 0.2 or 0.4 g glycine/kg body weight twice daily between 0 and 14 days of age. Alanine was used as the isonitrogenous control. At 14 days of age, the kidney was obtained from 6 pigs in each treatment group (see Table 3). The tissue was analyzed for free amino acids. Data were analyzed by one-way ANOVA and the Student-Newman-Keuls multiple comparison test. [a-d]: Within a row, means not sharing the same superscript letter differ ($p < 0.05$).

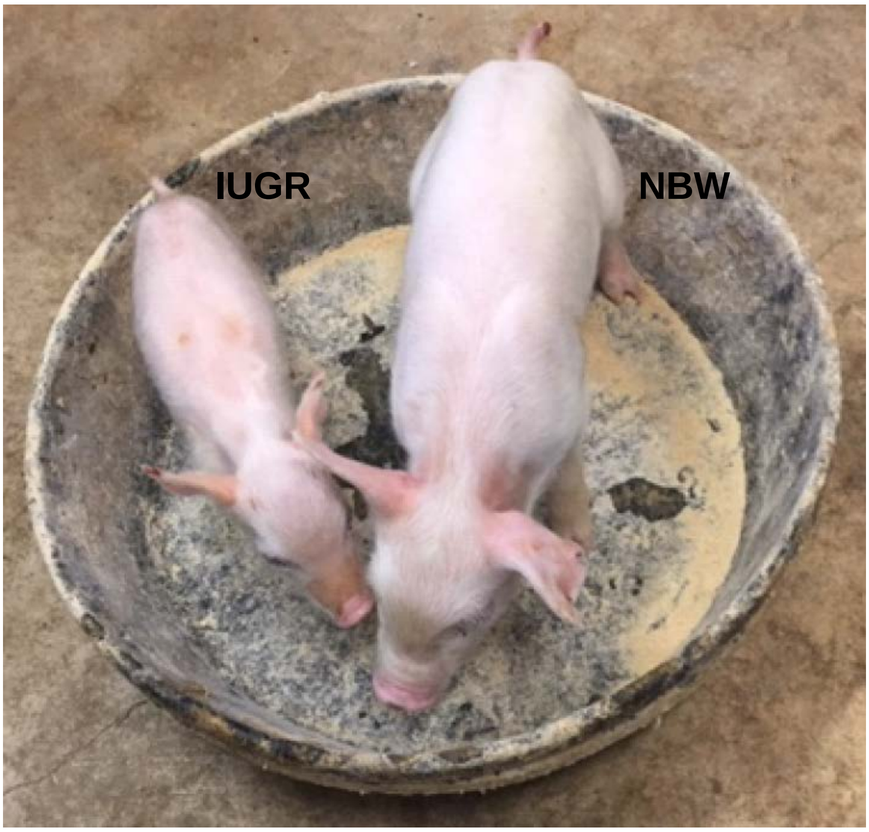

Supplementary Figure S1. Littermate piglets with intrauterine growth restriction (IUGR) and normal birth weight (NBW) at 14 days of age. At birth, runt piglets may weigh only one half or even one third as much as their largest littermates.
